# Heat and moisture exchangers (HMEs) and heated humidifiers (HHs) in adult critically ill patients: a systematic review, meta-analysis and meta-regression of randomized controlled trials

**DOI:** 10.1186/s13054-017-1710-5

**Published:** 2017-05-29

**Authors:** Maria Vargas, Davide Chiumello, Yuda Sutherasan, Lorenzo Ball, Antonio M. Esquinas, Paolo Pelosi, Giuseppe Servillo

**Affiliations:** 1 Department of Neurosciences, Reproductive and Odonthostomatological Sciences, University of Naples “Federico II”, Naples, Italy; 2 Dipartimento di Emergenza – Urgenza, ASST Santi Paolo e Carlo; Dipartimento di Scienze della salute, Università degli Studi di Milano, Milan, Italy; 3 Division of pulmonary and critical care medicine, Faculty of medicine Ramathibodi hospital, Mahidol University, 270 RAMA VI road, Bangkok, 10400 Thailand; 4 Department of Surgical Sciences and Integrated Diagnostics, AOU IRCCS San Martino- IST, University of Genoa, Genoa, Italy; 5 Intensive Care Unit, Hospital Morales Meseguer, Murcia, Spain

**Keywords:** Heated humidifiers, Heat and moisture exchangers, Artificial airway occlusion, Pneumonia, Mortality

## Abstract

**Background:**

The aims of this systematic review and meta-analysis of randomized controlled trials are to evaluate the effects of active heated humidifiers (HHs) and moisture exchangers (HMEs) in preventing artificial airway occlusion and pneumonia, and on mortality in adult critically ill patients. In addition, we planned to perform a meta-regression analysis to evaluate the relationship between the incidence of artificial airway occlusion, pneumonia and mortality and clinical features of adult critically ill patients.

**Methods:**

Computerized databases were searched for randomized controlled trials (RCTs) comparing HHs and HMEs and reporting artificial airway occlusion, pneumonia and mortality as predefined outcomes. Relative risk (RR), 95% confidence interval for each outcome and *I* ^2^ were estimated for each outcome. Furthermore, weighted random-effect meta-regression analysis was performed to test the relationship between the effect size on each considered outcome and covariates.

**Results:**

Eighteen RCTs and 2442 adult critically ill patients were included in the analysis. The incidence of artificial airway occlusion (RR = 1.853; 95% CI 0.792–4.338), pneumonia (RR = 932; 95% CI 0.730–1.190) and mortality (RR = 1.023; 95% CI 0.878–1.192) were not different in patients treated with HMEs and HHs. However, in the subgroup analyses the incidence of airway occlusion was higher in HMEs compared with HHs with non-heated wire (RR = 3.776; 95% CI 1.560–9.143). According to the meta-regression, the effect size in the treatment group on artificial airway occlusion was influenced by the percentage of patients with pneumonia (β = -0.058; *p* = 0.027; favors HMEs in studies with high prevalence of pneumonia), and a trend was observed for an effect of the duration of mechanical ventilation (MV) (β = -0.108; *p* = 0.054; favors HMEs in studies with longer MV time).

**Conclusions:**

In this meta-analysis we found no superiority of HMEs and HHs, in terms of artificial airway occlusion, pneumonia and mortality. A trend favoring HMEs was observed in studies including a high percentage of patients with pneumonia diagnosis at admission and those with prolonged MV. However, the choice of humidifiers should be made according to the clinical context, trying to avoid possible complications and reaching the appropriate performance at lower costs.

**Electronic supplementary material:**

The online version of this article (doi:10.1186/s13054-017-1710-5) contains supplementary material, which is available to authorized users.

## Background

Mechanical ventilation (MV) suppresses the mechanisms that heat and moisturize inhaled air. As a consequence, the lack of adequate conditioning may thicken airway secretions, which increases the airway resistance, reduces the gas exchange effectiveness and increases the risk of respiratory infections [1]. For these reasons, gas delivered during MV must be warmed and humidified to avoid serious complications related to dry gases [2]. To date, humidification devices can be divided into active heated humidifiers (HHs), which are devices heated by warm water, and passive devices such as heat and moisture exchangers (HMEs), which capture the heat of exhaled air and release it at the next inspiration [3]. HHs may result in increased airway hydration, decreased incidence of bacterial infection and work of breathing, while HMEs may increase the risk of airway occlusion [4]. In clinical practice, humidification during MV is widely accepted and applied; however, there is lack of consensus on the optimal device to humidify the airways. The aims of this systematic review and meta-analysis of randomized controlled trials are to evaluate the effects of HMEs and HHs in preventing artificial airway occlusion and pneumonia, and on mortality in adult critically ill patients. We planned a priori a sub-analysis stratifying the studies according to the type of HH, hypothesizing that HHs with heated wire could perform differently from those with non-heated wire. In addition, we planned to perform a meta-regression analysis to evaluate the relationship between the incidence of artificial airway occlusion, pneumonia and mortality and clinical features of adult critically ill patients.

## Methods

### Data sources and searches

We aimed to identify all randomized controlled trials (RCTs) comparing HMEs and HHs in adult critically ill patients. We applied standard filters for the identification of RCTs using the MEDLINE and PUBMED search engines (from inception to June 2014), using English language restrictions. Our search included the following keywords: *heat and moisture exchangers, heated humidifiers, airway humidification, artificial humidification, artificial airway occlusion, mortality, pneumonia and humans and randomized clinical trial.*

### Selection of studies

Trials comparing any type of HH, including systems with heated and non-heated wire, with HMEs in adult critically ill patients were included. We restricted the analysis to RCTs to guarantee control of selection bias. We included only published full papers and excluded abstracts. Study designs containing inadequately adjusted planned co-interventions and crossover trials were excluded. The intervention of interest was the use of HH and HME in reducing artificial airway occlusion, pneumonia and mortality. Studies were further divided according to the use of HH with heated and HH with non-heated wire to perform the subgroup analysis.

### Outcome measures

The primary outcome was the incidence of artificial airway occlusion; the secondary outcomes were the incidences of pneumonia and mortality.

### Data extraction and quality assessment

Initial selection was performed by two pairs of independent reviewers (MV and DC, PP and YS) screening titles and abstracts. For detailed evaluation, a full-text copy of all studies of possible relevance was retrieved. Data from each study were extracted independently by paired and independent reviewers (LB and DC, PP and YS) using a standardized data abstraction form. Data extracted from the publications were independently checked for accuracy by two other reviewers (GS and AE). Quality assessment of these studies included: (1) use of randomization sequence generation, (2) reporting and type of allocation concealment, (3) blinding, (4) reporting of incomplete outcome data and (5) comparability of the groups at baseline. Quality assessment is reported in Additional file 1. Two reviewers (MV and LB) independently used these criteria to evaluate trial quality. We solved any possible disagreement by consensus in consultation with two other reviewers (GS and AE) if needed.

### Qualitative analysis

A narrative summary approach was used to explore study characteristics and quality indicators in describing variation among studies and to consider possible implications for this in our understanding of the outcomes of the RCTs included in the Cochrane review [5, 6].

### Quantitative analysis

The meta-analysis was conducted according to the Preferred Reporting Items for Systematic Reviews and Meta-analyses (PRISMA) guidelines [7]. Meta-analysis was performed with mixed random effect using the DerSimonian and Laird method. Results were graphically represented using forest plot graphs. The relative risk (RR) and 95% CI for each outcome were separately calculated for each trial, pooling data when needed, according to an intention-to-treat principle. The choice to use RRs was driven by the design of meta-analysis based on RCTs. Tau^2^ was used to define the variance between studies. The difference in the estimates of treatment effect between the treatment groups for each hypothesis was tested using the two-sided *z* test with statistical significance considered at *p* value <0.05. The homogeneity assumption was checked with the *Q* test with a degree of freedom (df) equal to the number of analyzed studies minus 1. The heterogeneity was measured by the *I* ^2^ metric, which describes the percentage of total variation across studies that is due to heterogeneity rather than chance. *I* ^2^ was calculated as:

*I* ^2^ = 100% Å ~ (Q − df)/Q

where *Q* is Cochran’s heterogeneity statistic and df is degrees of freedom. A value of 0% indicates no observed heterogeneity, and larger values show increasing heterogeneity. We decided a priori to analyze all the outcomes according to the following categories when possible: HME vs HH, HME vs HH with heated wire and HME vs HH with non-heated wire.

Weighted random-effect meta-regression analysis was performed to test the relationship between the effect size on each considered outcome and the following covariates, with each one analysed separately: duration of MV, pneumonia incidence, intensive care unit (ICU) length of stay, percentage of respiratory diagnoses at ICU admission, simplified acute physiology score (SAPS), age and acute physiology and chronic health evaluation II (APACHE II) score.

Analyses were conducted with OpenMetaAnalyst (version 6) and SPSS version 20 (IBM SPSS). Weighted linear regression was used to evaluate potential publication bias, with the natural log of the RR as the dependent variable and the inverse of the total sample size as the independent variable. This is a modified Macaskill test that gives more balanced type I error rates in the tail probability areas in comparison to other publication bias tests [8].

## Results

### Study selection

**Fig. 1:**
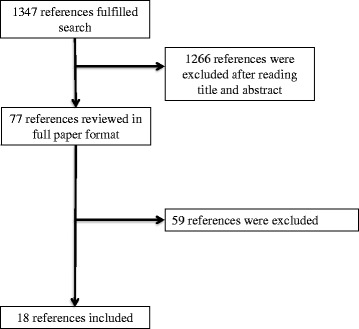
The study selection process

We identified 1349 references and excluded 1266 after screening titles and abstracts. We analyzed 77 articles in full paper format. We excluded 59 references [9–65] and 18 references fulfilled our search criteria [66–83]. Figure 1 shows the study selection process.

### Characteristics of included studies

These 18 RCTs included 2442 adult critically ill patients. The main characteristics of the included studies are reported in Table 1.

**Table 1:** Main characteristics of the randomized controlled trials included in the meta-analysis (HME vs HH with heated and non-heated wire)

First Author/year	Study design	Population	Age	Exclusion criteria	Number of patients (HME/HH)	Severity of illness	Characteristic of passive humidifier/frequency of change	Active humidifier (HH)	TV/MV	Frequency of change of ventilator circuit	Diagnosis of VAP
Oğuz 2013 [83]	SC/RCT	General ICU with intubation <24 hours	47.9 vs 44.5	Patients with intubation >24 hours, pneumonia	18 vs 17	n.a.	HME replaced daily	HH	n.a.	n.a.	CXR infiltration
Boots 2006 [82]	SC/RCT	General ICU with MV >48 hours	59 vs 60	Patients presenting history (airway hemorrhage, asthma, or airway burns) suggested a need for HH	190 vs 191	APACHE II 20 vs 20	Hygroscopic HME with a bacterial viral filter/24 hours	Hot-water humidification with a heated wire in both inspiratory and expiratory circuit limbs (DHW) or the inspiratory limb only (SHW)	n.a.	Every new patients	CPIS ≥6 Tracheal suction
Lorente 2006 [81]	SC/RCT	ICU with patients expected to require mechanical ventilation for >5 days	56 vs 55	Age <18 years, HIV, WBC <1000 cells/mm^3^ solid or hematological tumor and immunosuppressive therapy	53 vs 51	APACHE II 18.11/18.72	HME: Edith Flex (Datex-Ohmeda) changed at 48-hour interval	MR 850 ® (Fisher & Paykel Health Care Ltd, Auckland, New Zealand) and the Aerodyne 2000®servo-controlled humidifiers with wire-heated circuits without water traps and with an autofeed chamber to refill the chamber with water	n.a.	No routine change of ventilator circuit	Tracheal aspirate
Lacherade 2005 [80]	MC/RCT	5 ICUs located in two French university-affiliated teaching hospitals Medical, Surgical, Neurosurgical requiring MV >48 hours	55.2 vs 54.7	Contraindications to the use of an HMEF or of an HH, patients admitted after cardiac arrest, patients already enrolled in a clinical trial, and patients with early decision of treatment withdrawal were not included	185 vs 184	SAPS II 45.4 vs 49.3	DAR Hygrobac filter device (Tyco Healthcare/Nellcor, Pleasanton, CA, USA (changed at 48 hours interval)	The MR730 device (Fisher & Paykel Healthcare Ltd, Auckland, New Zealand). Heated wire	n.a.	Changed for every new patient	Invasive respiratory secretion samplings cultured quantitatively, using a protected telescoping catheter or BAL
Diaz 2002 [79]	SC/RCT	Intubated patients	61 vs 66	Previous pulmonary disease, hypothermia, pulmonary secretion or low expiratory volume	23 vs 20	n.a.	HME	HH	n.a.	n.a	n.a.
Memish 2001 [78]	SC/RCT	MV for 48 hours in adult ICUs, Medical surgical unit	47.7 vs 46	Ventilated <48 hours	123 vs120	APACHE II 20.8 vs 20.6	HME Hudson RCI, Temecula, CA, USA)/n.a.	HH	n.a.	n.a.	Tracheal aspirate
Kollef 1998 [76]	SC/RCT	17 years and required mechanical ventilation while in the ICU setting.	57.8 vs 59	Transferred from other hospitals and had already received mechanical ventilation, if they had heart or lung transplantation, or if they had massive hemoptysis	163 vs 147	APACHE II 17 vs 18.2	Nellcor Puritan-Bennett; Eden Prairie, Minn)/every week	HH with heated wire circuit	The number of patients requiring a minute ventilation >10 L/min (38% vs 34%)	Changed for every new patient	Tracheal aspirate
Lucchetti 1998 [77]	SC/RCT	Critically ill patients with mechanical ventilation	57 vs 56.3	n.a.	15 vs 30	n.a.	Hygrobac DAR	Bennett Cascade II, MR600 Fysher and Paykel set at 37 °C	TV 563 vs 594.2	n.a.	Airway secretion score
Boots 1997 [73]	SC/RCT	General/patients requiring MV > 48 hours	51	Patients with asthma, airway burns, or pulmonary hemorrhage	42 (2 days), 33 (4 days) vs HH 41 (2 days)	APACHE II 19 vs18	Bacterial-viral filter (Humid-Vent Filter Light, Gibeck Respiration, Vasby, Sweden)/2 days or 4 days circuit change (2 groups)	MR730, Fisher and Paykel Health Care Pty Ltd, Auckland, New Zealand/HH circuit with 2 days circuit change	n.a.	Every 48 hours	Tracheal aspirate
Hurni 1997 [74]	SC/RCT	Medical ICU/patients who required >48 hours of MV	52.6 vs 59.5	Hypothermic (central or rectal temperature <36 °C), or who had been intubated for 12 hours before ICU admission were excluded	59 vs 56	SAPS II 12.9 vs 12.8	Hygroster; DAR; Mirandola, Italy/every 24 hours	Fisher Paykel; Auckland, New Zealand, or Puritan-Bennett set at 37 °C	n.a.	48 Hours in HH group and weekly in HME	Tracheal aspirate
Kirton 1997 [75]	SC/RCT	20-Bed trauma ICU >15 years who required MV	47/46 vs 48	Yes: requirement for high minute volume	280	Injury severity score (ISS) 22 vs 20	Pall BB-100; Pall Corporation; East Hills, NY, USA (hydrophobic) 24 hours	Heated wire humidifier (H-wH) (Marquest Medical Products Inc., Englewood, CO, USA)	n.a.	Every 7 days	Tracheal aspirate
Branson 1996 [71]	SC/RCT	Surgical-medical ICU patients requiring mechanical ventilation deemed suitable for HME	44 vs 41	Patients deemed unsuitable for HME such as presence of thick or bloody secretions	49 vs 54	SAPS II 9 vs 8	HME hygroscopic Baxter/24 hours	Heated wire humidifier MR730 (Fisher & Paykel) set at 36 °C	n.a.	Every 7 days	Tracheal aspirate
Villafane 1996 [72]	SC/RCT	Intubated and mechanically ventilated patients	67 vs 59	Patients with hemorrhagic disorder, intubated >24 hours, expected for intubation for short time, drugs overdose	16 vs. 7	SAPS 17 vs 17	HME hygroscopic BB-2215, Pall. HME Hygroscopic 352/5411 DAR	MR310 Fysher and Paykel set at 32 °C	MV 11.3 vs 10.2 L/min	n.a.	n.a.
Dreyfuss 1995 [70]	SC/RCT	Medical patients who required >48 hours of MV	58 vs 62	No	61 vs 70	SAPS II 16.0 vs 16.4	HME hygroscopic DAR-Hygrobac II (DAR SpA, Mirandola, Italy) device three-layer water-repellent membranes with electrostatic and mechanical filtering power and of one hygroscopic membrane/change on daily basis	Puritan-Bennett Respiratory Products, Santa Monica, CA) or Fischer-Paykel MR 450 or MR 460 devices	n.a.	Every new patient	Quantitative cultures of protected specimen brush
Roustan 1992 [69]	SC/RCT	General/patients requiring MV (France)	52.7 (18.5) vs 49.3 (18.7)	Weight less than 35 kg and patients requiring high-frequency jet ventilation	55 vs 61	SAPS II 11.5 vs 11.5	Pall Filter BB 2215 HME (hygrophobic)/every 24 hrs	Draegger Aquaport, temperature was set 31 and 32 at the Y piece.	TV 665 vs 460 ml	n.a.	None
Misset 1991 [68]	SC/RCT	Medical-surgical/patients requiring MV >5 days (France)	53(14) vs 49 (13)	No	30 vs 26	SAPS II 14 vs 13	HME hydrophobic (every 24 hours)	Bennett cascade II or Fisher Paykel MR 450 set at 32 °C or 34 °C	No difference in tracheal thickness and characteristic between MV >10 L and <10 L 11.9 (2.5) vs 11.2 (2.9)	Every 48 hours	Tracheal aspirate
Martin 1990 [67]	SC/RCT	All patients to receive mechanical ventilation for more than 24 hours	61 vs 54	No	31 vs 42	n.a.	Pall Ultipor (hydrophobic) breathing circuit filter (PUBCF) replaced at least daily	HH: set at 31 °C	11 (2.5) vs 10.11 (3)	3 Times weekly	Tracheal aspirates
Kirkegaard 1987 [66]	SC/RCT	Neurosurgical patients	15 vs 15	No	52 vs 36	n.a.	HME hygroscopic Engstrom Edith, Gambro	HH Hygrotherm	n.a	24 Hours	None

*HME* heat and moisture exchanger, *HH* heated humidifier, *MV* mechanical ventilation, VAP, ventilator-associated pneumonia, *RCT* randomized controlled trial, *WBC* white blood cells, *APACHE* acute physiology and chronic health evaluation, *SAPS* simplified acute physiology score

### Systematic errors of included studies

None of the included trials had a low risk of bias. The random sequence generation was adequate in seven studies [75, 78–83], adequate allocation concealment was present in three studies [77–79] and adequate blinding was present in two studies [75, 76]. Complete outcome data were reported from 10 studies [66–70, 73–76, 80–83]. Ten studies reported no imbalance in baseline characteristics [66–71, 73, 76, 79, 81]. The quality assessment for each RCT is reported in Additional file 1.

### Primary outcome

**Fig. 2:**
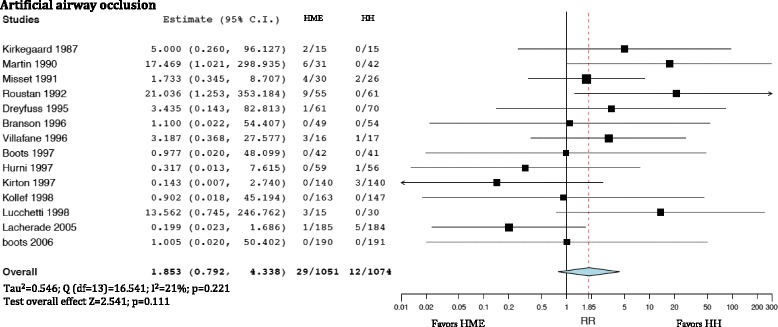
Artificial airway occlusion comparing the heat and moisture exchanger (*HME*) with the heated humidifier (*HH*). Weights: Kirkegaard 6.8%, Martin 7.1%, Misset 15.3%, Roustan 7.1%, Dreyfuss 5.9%, Branson 4.2%, Villafane 10.7%, Boots (2006) 4.2%, Hurni 5.9%, Kirton 6.6%, Kollef 4.1%, Lucchetti 6.9%, Lacherade 10.8%, boots (1997) 4.1%

**Fig. 3:**
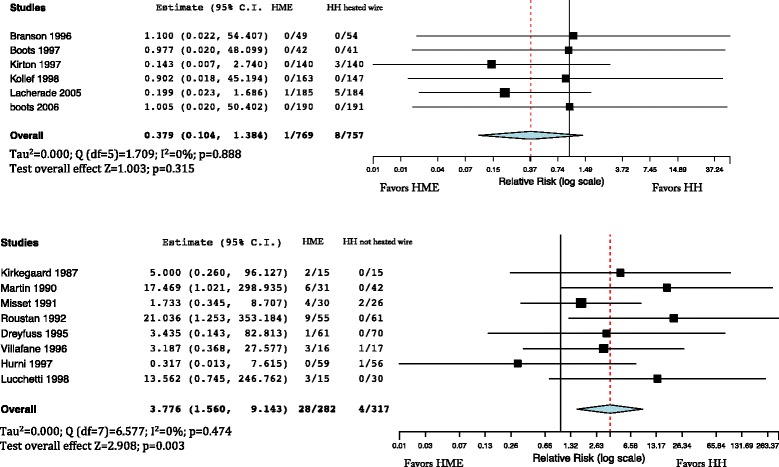
*Upper box* artificial airway occlusion comparing the heat and moisture exchanger (HME) and the heated humidifier (HH) with heated wire. Weights: Branson 11%, Boots (1997) 11%, Kirton 19.2%, Kollef 11%, Lacherade 36.8% Boots (2006) 10.9%. *Lower box* artificial airway occlusion comparing HME and HH with non-heated wire. Weights: Kirkegaard 8.9%, Martin 9.7%, Misset 30%, Roustan 9.8%, Dreyfuss 7.7%, Villafane 16.8%, Hurni 7.7%, Lucchetti 9.3%

The incidence of artificial airway occlusion was not different in patients treated with HMEs and HHs (Fig. 2) (RR = 1.853; 95% CI 0.792–4.338). Artificial airway occlusion incidence was not different when comparing HMEs with HHs with heated wire (RR = 0.379; 95% CI 0.140–1.384) (Fig. 3, upper panel). However, airway occlusion was higher with HMEs compared with HHs with non-heated wire (RR = 3.776; 95% CI 1.560–9.143) (Fig. 3, lower panel), but there were no differences between hydrophobic and hygroscopic HMEs compared with HHs (Additional file 2).

### Secondary outcomes

**Fig. 4:**
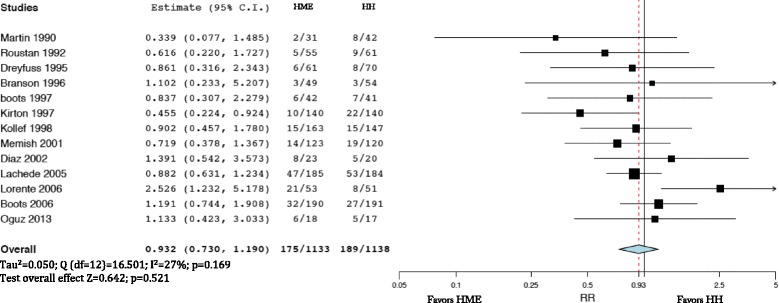
Incidence of pneumonia comparing the heat and moisture exchanger (*HME*) with the heated humidifier (*HH*). Weights: Martin 2.5%, Roustan 4.7%, Dreyfuss 5%, Branson 2.3%, Boots (1997) 5%, Kirton 8.6%, Kollef 9.1%, Memish 9.4%, Diaz 5.5%, Lacherade 19.5%, Lorente 8.4%, Boots (2006) 14.3%, Oguz 5.1%

**Fig. 5:**
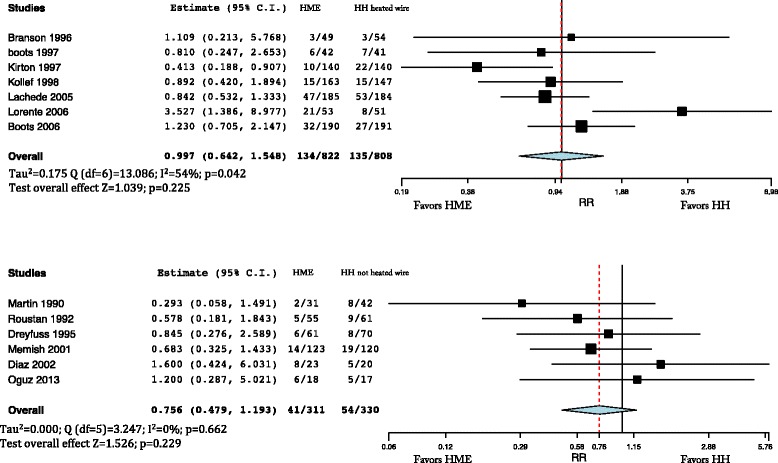
*Upper panel*: incidence of pneumonia comparing the heat and moisture exchanger (*HME*) and the heated humidifier (HH) with heated wire. Weights: Branson 5.7%, Boots (1997) 9.3%, Kirton 15%, Kollef 15.7%, Lachede 22%, Lorente 12.6%, Boots (2006) 19.7%. *Lower panel* incidence of pneumonia comparing HME and HH with non-heated wire. Weights: Martin 7.8%, Roustan 15.5%, Dreyfuss 16.7%, Memish 37.9%, Diaz 11.8%, Oguz 10.2%

The incidence of pneumonia was not different in patients treated with HMEs and HHs (Fig. 4) (RR = 932; 95% CI 0.730–1.190). Incidence of pneumonia was not different when comparing HMEs and HHs with heated wire (RR = 0.997; 95% CI 0.642–1.548), with significant inhomogeneity (*I* ^2^ = 54%; *p* = 0.042) (Fig. 5, upper panel), neither was it different with HHs with non-heated wire (RR = 0.756; 95% CI 0.479–1.193) (Fig. 5, lower panel).

**Fig. 6:**
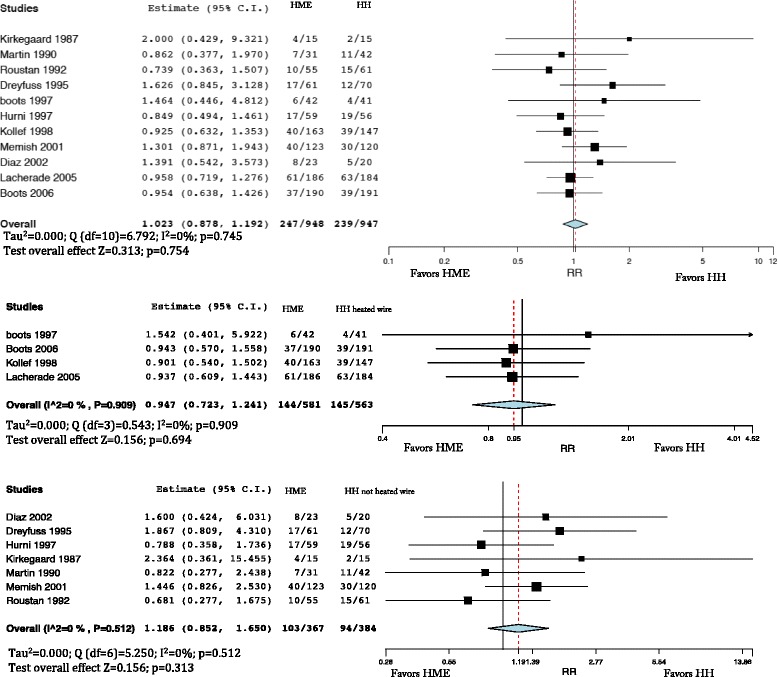
*Upper panel* mortality comparing the heat and moisture exchanger (*HME*) with the heated humidifier (*HH*). Weights: Kirkegaard1%, Martin 3.4%, Roustan 4.6%, Dreyfuss 5.4%, Boots (1997) 1.6%, Hurni 7.9%, Kollef 16.1%, Memish 14.5%, Diaz 2.6%, Lacherade 28.3%, Boots (2006) 14.4%. *Middle panel* mortality comparing HME and HH with heated wire. Weights: Boots (1997) 4%, Boots (2006) 28.9%, Kollef 27.9%, Lacherade 39.2%. *Lower box* mortality comparing HME and HH with non-heated wire. Weights: Diaz 6.2%, Dreyfuss 15.6%, Hurni 17.51%, Kirkegaard 3.1%, Martin 9.2%, Memish 34.9%, Roustan 13.5%

Mortality was not different in patients treated with HMEs and HHs (Fig. 6, upper panel) (RR = 1.023; 95% CI 0.878–1.192). Mortality was comparable in patients treated with HMEs and HHs with heated wire (RR = 0.947; 95% CI 0.723–1.241) (Fig. 6, middle panel). We did not find differences in mortality when comparing HMEs and HHs with non-heated wire (RR = 1.186; 95% CI 0.852–1.650) (Fig. 6, lower panel).

### Meta-regression analysis

**Fig. 7:**
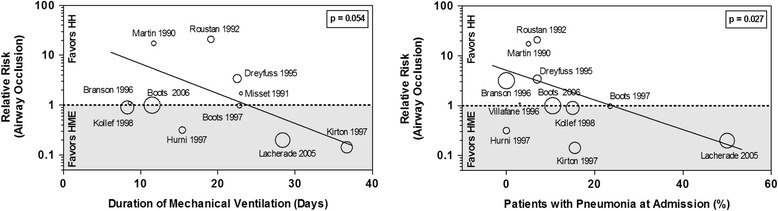
Meta-regression on artificial airway occlusion including duration of mechanical ventilation (β = 2.637; *p* = 0.054) and pneumonia (β = 1.794; *p* = 0.012) as covariate. *HME* heat and moisture exchanger, *HH* heated humidifier

The effect size in the treatment group on artificial airway occlusion was influenced by the percentage of patients with pneumonia included in the study (β = -0.058; *p* = 0.027; favoring HMEs in studies with high prevalence of pneumonia), and a trend was observed for the duration of MV (β = -0.108; *p* = 0.054; favoring HMEs in studies with longer MV time) (Fig. 7). No other significant associations with the effect size on any outcome measure were observed for the other clinical variables (see Additional file 3).

## Discussion

In this systematic review and meta-analysis, we found: (1) no significant difference in artificial airway occlusion, pneumonia or mortality between HMEs and HHs, (2) no effect of HHs with and without heated wire compared to HMEs; however HHs with non-heated wire had the lower RR for artificial airway occlusion compared with HME, and (3) independently from the HH type, an advantage of HMEs in airway occlusion incidence was observed in studies with high incidence of pneumonia, and a trend toward favoring HMEs was observed for studies with prolonged MV. To our knowledge, this is the first systematic review performed (1) by dividing RCTs according to HHs with heated and non-heated wire and (2) including a meta-regression analysis on the potential effects of clinical variables on the efficacy of the two devices.

According to the American Association for Respiratory Care (AARC) guidelines, HHs should provide an absolute humidity level of between 33 and 44 mgH2O/L, whereas HMEs should provide a minimum humidity level of 30 mgH2O/L [1]. HHs may produce insufficient heat and humidification when the temperature is improperly selected or pre-set at a non-adjustable level rather than at the clinical setting [1]. However, insufficient heat and humidification may occur with HMEs too [1]. Only 37% of HMEs have been found to meet the standard criteria advocated by the AARC guidelines [1]. Insufficient airway humidification may lead to an increase in tracheal tube occlusion, a serious adverse event that may occur in mechanically ventilated patients and requires timely intervention. In an RCT comparing HMEs and HHs with increasing minute ventilation, the authors found that after 72 hours the inner diameter of the endotracheal tube decreased by 2.5–6.5 mm when gas conditioning was performed using HMEs and by 1.5 mm with HHs [72]. A systematic review showed that in patients ventilated more than 48 hours, there is no difference in tracheal tube occlusion when comparing HMEs and HHs [84]. In this meta-analysis, we found no differences in the incidence of artificial airway occlusion, but stratifying the comparison according to the type of HHs we found less risk for airway occlusion in HHs without heated wire compared with HME. However, these data were not confirmed by the sub-analysis comparing hydrophobic and hygroscopic HMEs with HHs. Probably, the main determinants of artificial airway occlusion are the duration of mechanical ventilation and pneumonia, rather than humidifier type per se*,* even if a prolonged use of HME (<72 hours) may increase this risk. Long-term invasive MV and the presence of ventilator-associated pneumonia (VAP) increased the risk of artificial airway occlusion threefold in one study [85] and twofold in another [86]. This is the first meta-analysis reporting a meta-regression of included studies in this field. Our meta-regression showed that in studies with high incidence of pneumonia and prolonged MV, the HMEs had a slight advantage in terms of the artificial airway occlusion.

Earlier models of HME were associated with an increased incidence of airway occlusion, which led to the exclusion of patients at high risk from the studies [86]. In contrast, trials using HMEs with enhanced intrinsic humidifying performance showed no difference in the incidence of airway occlusion [86]. A Cochrane review states that hydrophobic HMEs may reduce the risk of pneumonia and the use of an HME may increase artificial airway occlusion in certain subgroups of patients [4]. Our analysis includes more recently published studies. According to our meta-regression, the HME may reduce the risk of airway occlusion in selected patients affected by pneumonia.

Hess et al. concluded in their clinical practice guidelines that HMEs are associated with lower incidence of pneumonia compared with HHs [85]. However, there are concerns about the increased airway resistance and care of HME filters [85]. Kola et al. found a significant reduction in pneumonia using HMEs during MV, particularly when patients are ventilated for 7 days or more [86]. Hess et al. included studies published between 1990 and 1998 in their analysis of pneumonia [85]. Kola et al. reported the same results as Hess et al. but they only included one more study in their meta-analysis. Accordingly, the underlying mechanism of reduction in pneumonia may be due to the dryness of the ventilator circuit when using HMEs [85, 86]. Therefore, HMEs minimized the need for septic manipulations or aspirations of the airway/circuit and the circuit condensate [85, 86]. Furthermore, the inclusion of more recent studies may have changed the results. Indeed, Siempos et al. did not find any superiority of HMEs compared to HHs in reducing pneumonia, mortality or morbidity [87]. The results of Siempos et al. were groundbreaking and in line with the RCTs published at that time. The inclusion of three further RCTs [80–82] with 870 patients dramatically changed the previous results. The Cochrane review by Kelly et al. included adult and pediatric patients treated with HMEs and HHs [4]. There was no overall effect on artificial airway occlusion, mortality, pneumonia or respiratory complications; however, the arterial partial pressure of carbon dioxide (PaCO_2_) and minute ventilation were increased while body temperature was lower when HMEs were compared to HHs [4] in a meta-analysis including 18 RCTs and 2442 adult critically ill patients. In line with the available literature, we did not find any difference in artificial airway occlusion, pneumonia or mortality between HMEs and HHs, even if according to their nature, they have different characteristics. However, HMEs were found to increase the PaCO_2_ and work of breathing probably due to higher dead space, and to reduce the inner diameter of the endotracheal tube during prolonged MV [4]. Indeed, HME may negatively impact on ventilator function while increasing the dead space [11]. In spontaneously and assisted breathing patients, this requires increased minute ventilation and then the work of breathing, to maintain constant alveolar ventilation and PaCO_2_. In controlled MV, the additional dead space of HMEs may reduce alveolar ventilation and increase PaCO_2_ [11]. This effect of HME dead space may be further exacerbated by protective ventilation at low tidal volume (VT) and by using HMEs with a larger dead space [11].

Humidification is mandatory during MV. Nowadays, the airway humidification is appropriate in the absence of any contraindications listed by the AARC guidelines, such as altered body temperature, airway thermal injury, under hydrated secretions, increased work of breathing, hypoventilation, condensation and airway dehydration [1]. Clear advantages in terms of clinical outcomes for different humidification devices are far from being demonstrated. The present meta-analysis reported no superiority of HMEs over HHs in term of clinical outcomes, with similar results even when stratifying the studies according to the type of HH, while some advantage of HMEs might be possible in patients with pneumonia or those with a long MV time. The choice of humidifiers should be made according to the clinical context trying to avoid possible complications and reaching the appropriate performance at lower costs. However, to help clinicians make the correct choice between HHs and HMEs, further high-quality RCTs are needed to evaluate the incidence of respiratory complications other than pneumonia, gas exchange and work of breathing when comparing the HH and HME devices.

This systematic review and meta-analysis has several limitations that must be addressed. First, the quality of the included RCTs was relatively low and our conclusions may be limited by this point. Second, the diagnosis of pneumonia was differently defined across the studies and often mixed with VAP. Third, the definition of mortality varied between the studies: three studies reported the ICU mortality, two studies reported overall mortality, two studies reported hospital mortality, one studies reported mortality during MV and in four studies mortality was not reported. Fourth, we performed the meta-analyses of outcomes if reported by more than three RCTs. Fifth, as we found only one additional RCT published between 2006 and 2013, the present results may depend on the studies published before 2006. However, in contrast to previous reports we included a meta-regression analyzing and interpreting data from a new point of view. Sixth, we found few studies reporting the effective tracheal tube lumen and most of them with provided a poor definition of pneumonia diagnosed at ICU admission. Seventh, we are not able to stratify the meta-analysis according to the baseline respiratory condition or inclusion criteria. This did not allow us to suggest the use of HMEs or HHs in different respiratory diseases.

New, prospective RCTs are needed in terms of assessing the effect of HHs vs HMEs in patients with respiratory failure due to pneumonia, on airway diameter, the amount of secretion and the occurrence of artificial airway obstruction and VAP.

## Conclusions

In this meta-analysis including 18 RCTs and 2442 adult critically ill patients, we found no superiority of HMEs or HHs, in terms of artificial airway occlusion, pneumonia and mortality. These results were also confirmed in the sub-analysis dividing HHs into heated and non-heated wire devices. However, HHs with non-heated wire had the lower RR for artificial airway occlusion compared with HMEs. A trend favoring HMEs was observed in studies including a high percentage of patients with pneumonia diagnosis at admission and those with prolonged MV. However, the choice of humidifiers should be made according to the clinical context, trying to avoid possible complications and reaching the appropriate performance at lower costs.

## Additional files

Additional file 1: Quality assessment. Quality assessment of these studies included: 1) use of randomization sequence generation, 2) reporting and type of allocation concealment, 3) blinding, 4) reporting of incomplete outcome data, 5) comparability of the groups at baseline. H: high, L: low, U: unknown. (PDF 80 kb)

Additional file 2: Meta-regression. Meta-regressions for artificial airway occlusion, pneumonia and mortality. (PDF 411 kb)

Additional file 3: Meta-analyses for hydrophobic and hygroscopic HME vs HH for Artificial airway occlusion, mortality and Pneumonia. (PDF 231 kb)

## Abbreviations

AARC: American Association for Respiratory Care
APACHE II: Acute physiology and chronic health evaluation II
CI: Confidence interval
HHs: Heated humidifiers
HMEs: Heat and moisture exchangers
ICU: Intensive care unit
MV: Mechanical ventilation
PaCO_2_: Arterial partial pressure of carbon dioxide
RCTs: Randomized controlled trials
RR: Relative risk
SAPS: Simplified acute physiology score
VAP: Ventilator-associated pneumonia
