# Supplementary material for: Heat and moisture exchangers (HMEs) and heated humidifiers (HHs) in adult critically ill patients: a systematic review, meta-analysis and meta-regression of randomized controlled trials
Source: Crit Care. 2017 May 29;21:123. doi: 10.1186/s13054-017-1710-5 (PMC5447307; doi:10.1186/s13054-017-1710-5)
Supplement: Supplementary file 1 — Quality assessment. Quality assessment of these studies included: 1) use of randomization sequence generation, 2) reporting and type of allocation concealment, 3) blinding, 4) reporting of incomplete outcome data, 5) comparability of the groups at baseline. H: high, L: low, U: unknown. (PDF 80 kb) [file 13054_2017_1710_MOESM1_ESM.pdf]

	**Random sequence generation**	**Allocation concealment**	**Blinding**	**Incomplete outcome data**	**Baseline characteristics**
Oguz 2013	Random number list generated using Excel software<br>L	Not described<br>U	Not described<br>U	Not described<br>U	Imbalance in baseline characteristic (gender, smoking, chronic disease) and time of circuit changes<br>H
Boots 2006	Predetermined list generated from a list of random numbers.<br>L	Not described<br>U	Blinding only CXR<br>U	No lost to follow-up<br>L	Imbalance in baseline characteristics (Length of MV)<br>H
Lorente 2006	Random number list generated using Excel software<br>L	Not described<br>U	Not described<br>U	16/120 lost to follow-up<br>L	No imbalance in baseline characteristics<br>L
Lacherade 2005	Computer generated randomization<br>L	Not described<br>U	Not described<br>U	No lost to follow-up<br>L	Imbalance in baseline characteristics (P/F ratio and immunocompromised pts)<br>H
Diaz 2002	Random number list<br>L	Sealed and consecutively numbered<br>L	Not described<br>U	No lost follow up<br>H	Balance in baseline characteristics<br>L
Memish 2001	Group balance was maintained within each block of 20<br>L	The randomization record was kept with the hospital biostatistician<br>L	Not described<br>U	155/398 lost follow up<br>H	Imbalance in baseline characteristics (neurologic disease)<br>H
Kollef 1998	Not described<br>U	Opaque envelopes<br>L	Blinding assessment<br>L	Not described<br>U	Balance in baseline characteristics<br>L
Lucchetti 1998	Not described<br>U	Not described<br>U	Not described<br>U	Not described<br>U	Not described<br>U

Boots 1997	Not described U	Not described U	Not described U	No lost to follow-up L	Balance in baseline characteristics L
Hurni 1997	Not described U	Not described U	Not described U	50% lost to follow-up H	Imbalance in baseline characteristic(age) and time of circuit changes H
Kirton 1997	Randomization was by a random number generated from a personal computer L	Not described U	Laboratory and chest radiograph interpretation were blinded L	6/280 lost to follow up L	Not described U
Dreyfuss 1995	Not described U	Not described U	Not described U	36/164 lost to follow up L	Balance in baseline characteristics L
Branson 1996	Randomization was accomplished using the last digit in the patient's medical record number --- patients with an odd medical record number received an HCH and those with an even number received a heated humidifier H	Randomization was accomplished at the Bedside H	Not described U	Number enrolled not stated U	Balance in baseline characteristics L
Villafane 1996	Not described U	Not described U	Not described U	Not described U	Not described U
Rouston 1992	Not described U	Not described U	Not described U	100% follow up L	Balance in baseline characteristics L
Misset 1991	Not described U	Not described U	Not described U	18/74 lost to follow up L	Balance in baseline characteristics L

Martin 1990	Not described U	Not described U	Not described U	No lost to follow-up L	Balance in baseline characteristics L
Kirkegaard 1997	Not described U	Not described U	Not described U	No lost to follow-up L	Balance in baseline characteristics L

Table 2: description of quality assessment for each included study.
H= high risk of bias; L= low risk of bias; U= uncertain risk of bias,
