# Supplementary material for: Heat and moisture exchangers (HMEs) and heated humidifiers (HHs) in adult critically ill patients: a systematic review, meta-analysis and meta-regression of randomized controlled trials
Source: Crit Care. 2017 May 29;21:123. doi: 10.1186/s13054-017-1710-5 (PMC5447307; doi:10.1186/s13054-017-1710-5)
Supplement: Supplementary file 2 — Meta-regression. Meta-regressions for artificial airway occlusion, pneumonia and mortality. (PDF 411 kb) [file 13054_2017_1710_MOESM2_ESM.pdf]

META-REGRESSION FOR ARTIFICIAL AIRWAY OCCLUSION

No significant relationships were found between artificial airway occlusion and ICU LOS, % respiratory diagnoses, SAPS, APACHE II and age.

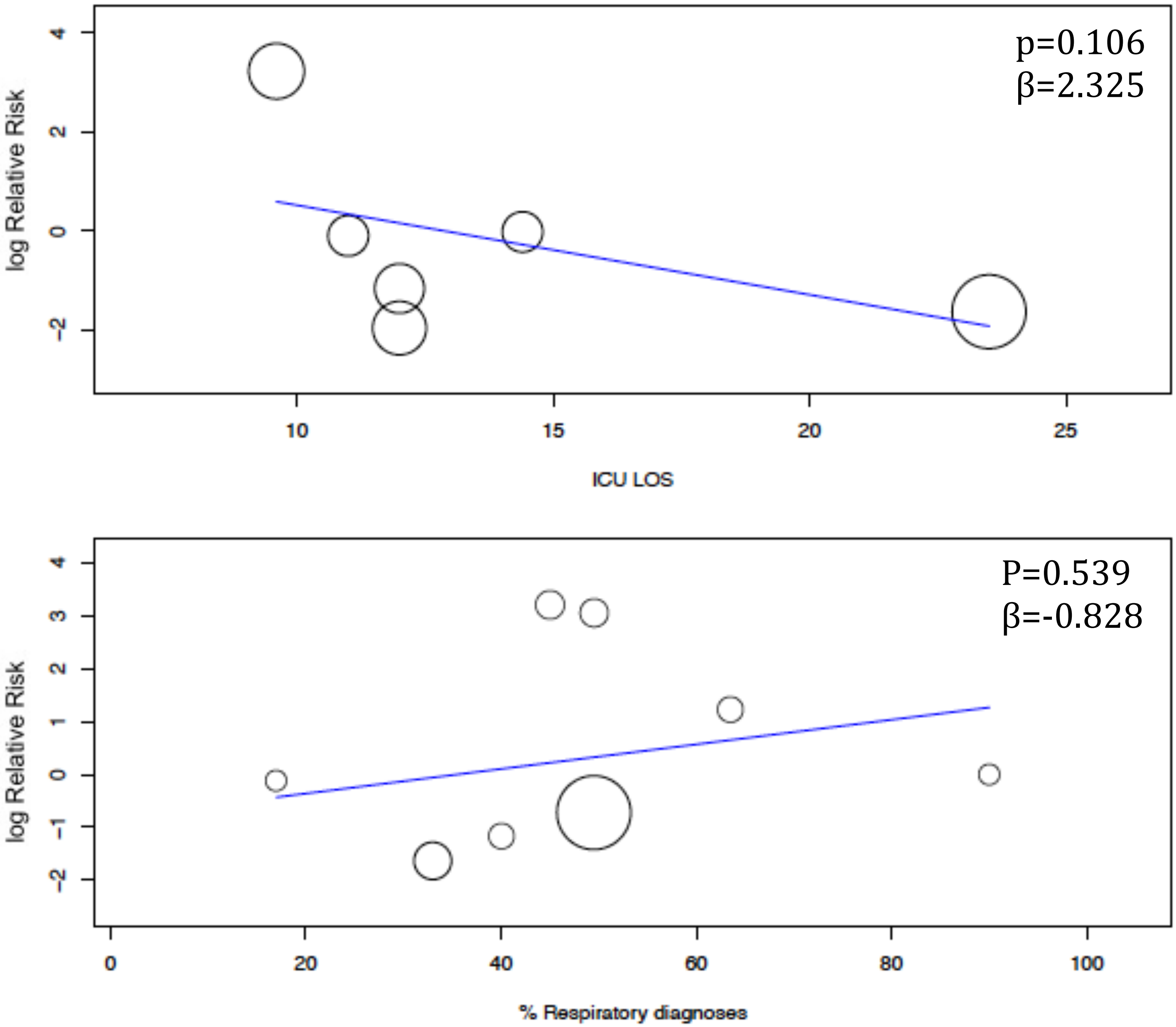
p=0.106
β=2.325
log Relative Risk
ICU LOS
P=0.539
β=-0.828
log Relative Risk
% Respiratory diagnoses

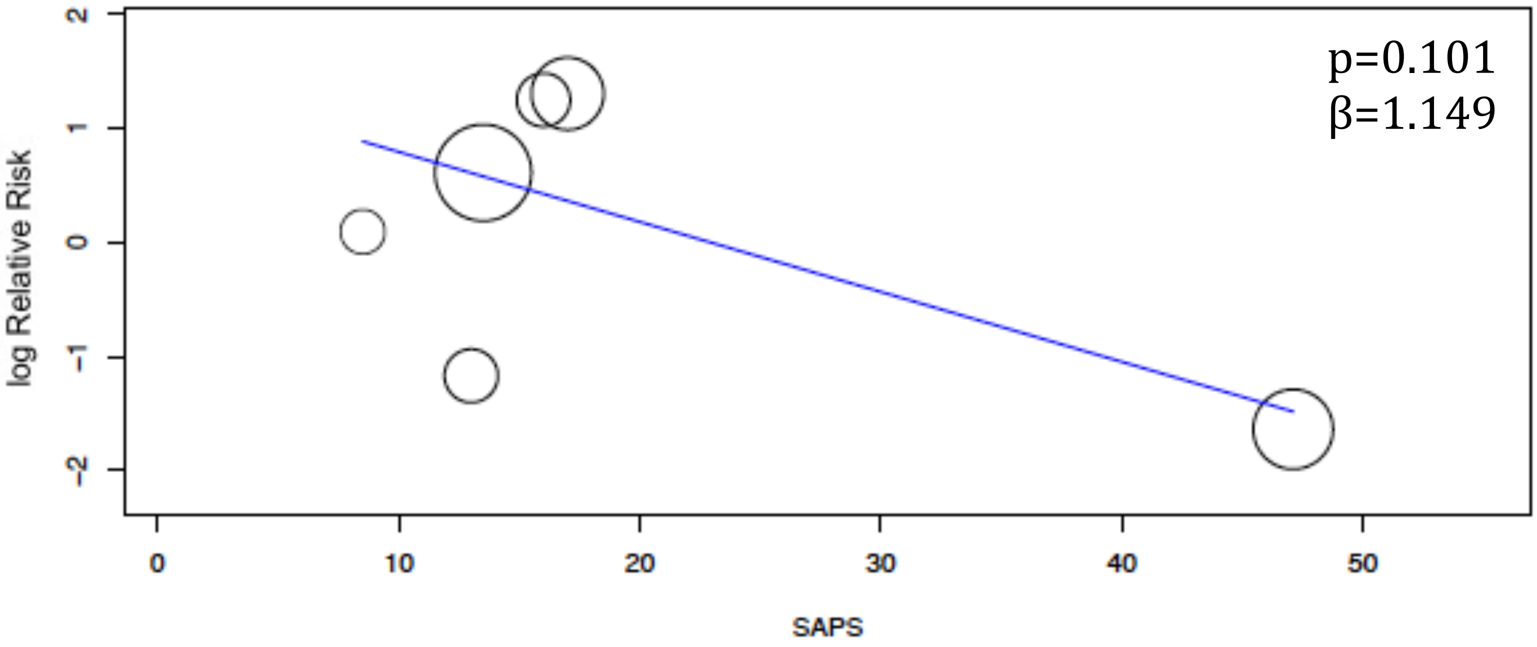
p=0.101
β=1.149
log Relative Risk
2
1
0
−1
−2
0
10
20
30
40
50
SAPS

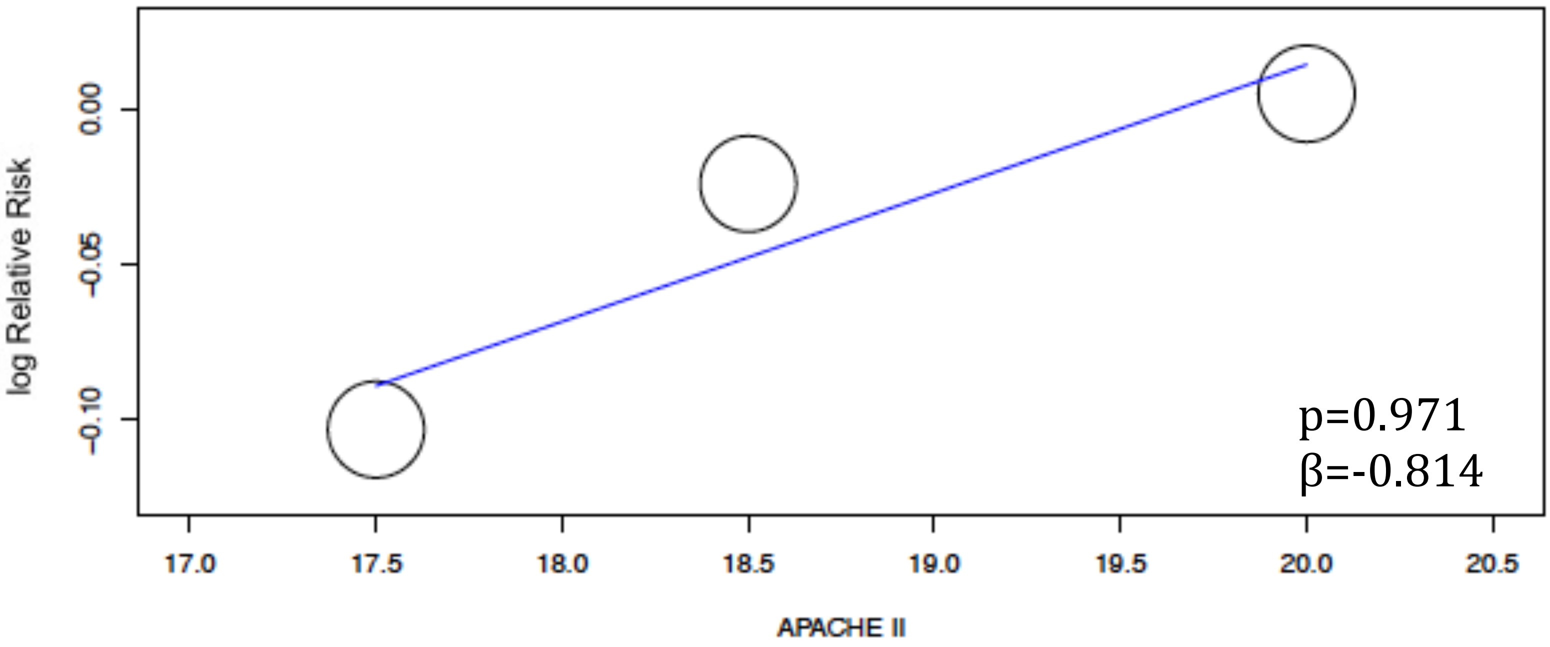
p=0.971
β=-0.814
log Relative Risk
0.00
−0.05
−0.10
17.0
17.5
18.0
18.5
19.0
19.5
20.0
20.5
APACHE II

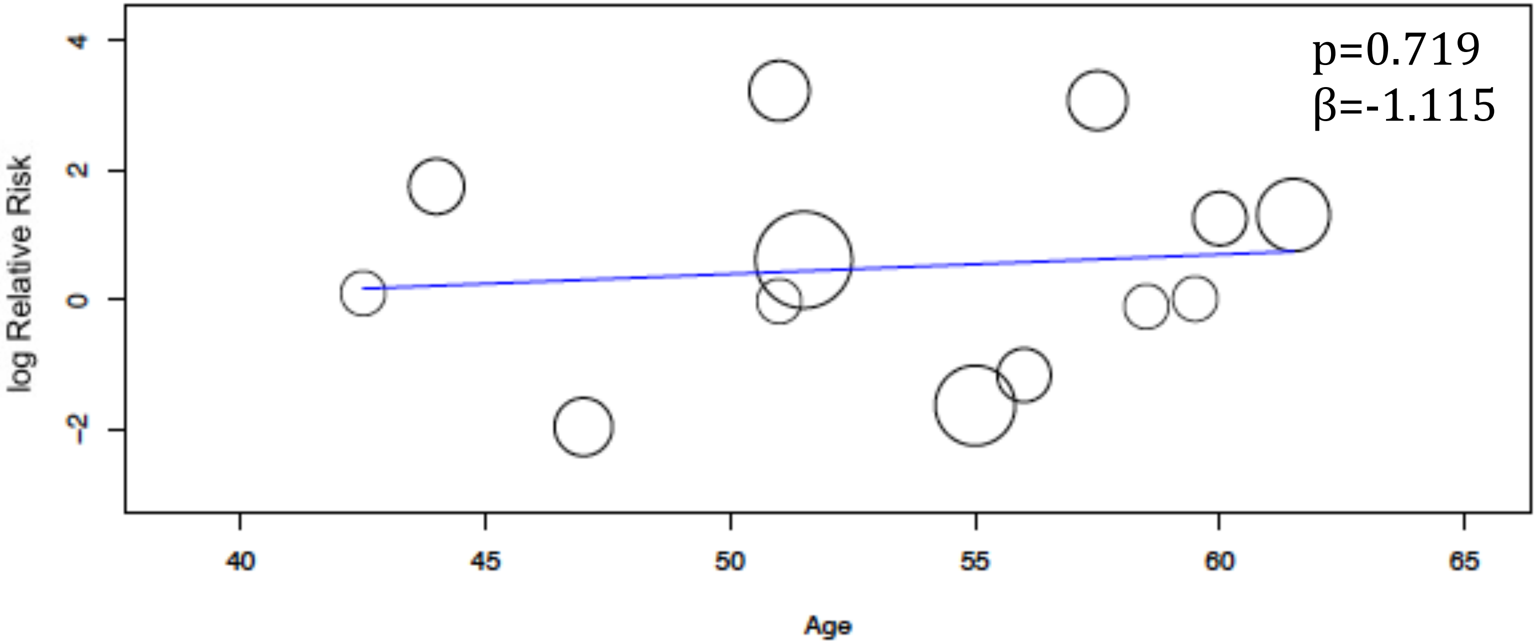
p=0.719
β=-1.115
log Relative Risk
4
2
0
-2
40
45
50
55
60
65
Age

META-REGRESSION FOR PNEUMONIA

No significant relationships were found between incidence of pneumonia and duration of MV, AIW occlusion/reintubation, ICU LOS, % respiratory diagnoses, SAPS, APACHE II and age.

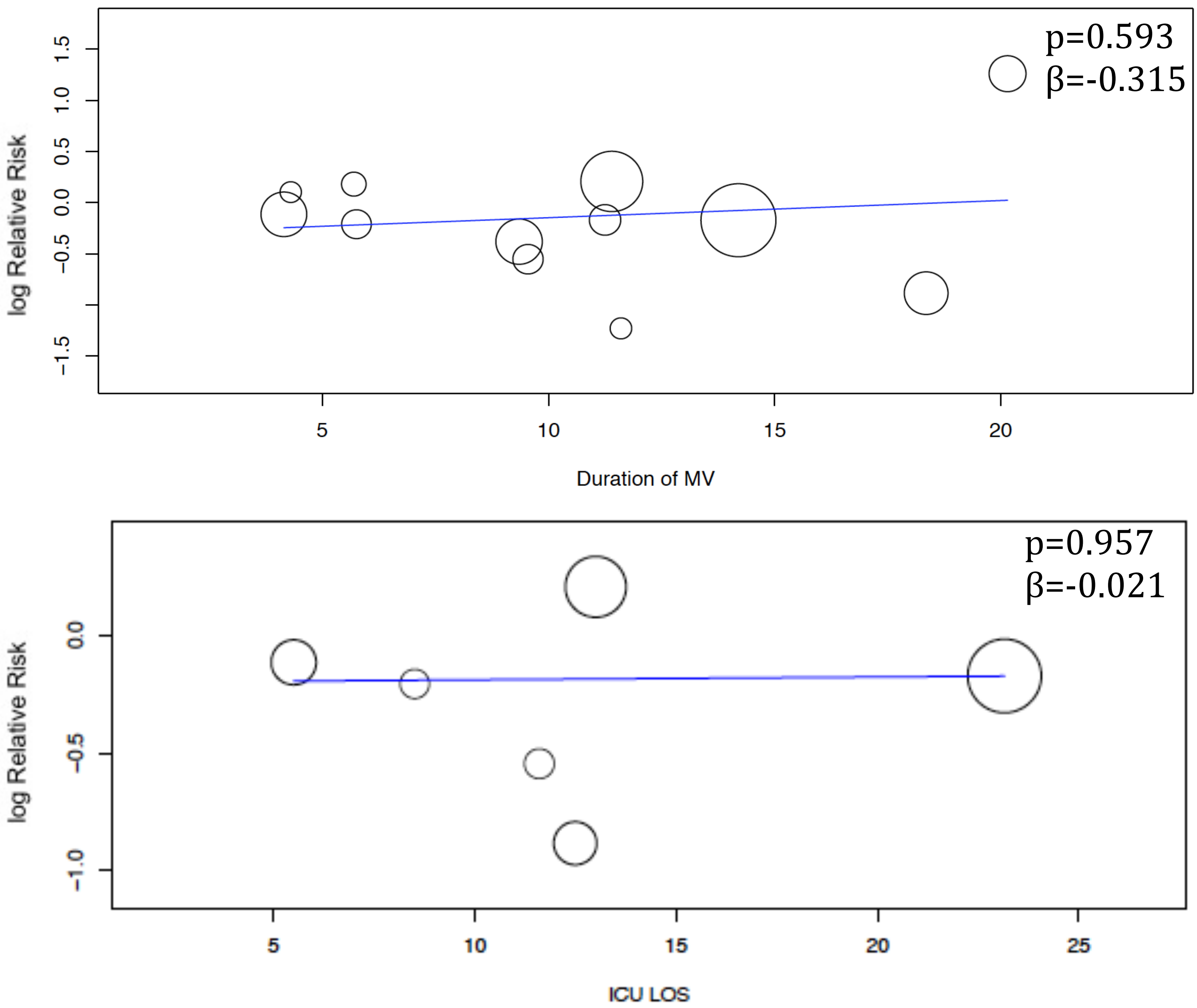
p=0.593
β=-0.315
log Relative Risk
1.5
1.0
0.5
0.0
−0.5
−1.5
5
10
15
20
Duration of MV
p=0.957
β=-0.021
log Relative Risk
0.0
−0.5
−1.0
5
10
15
20
25
ICU LOS

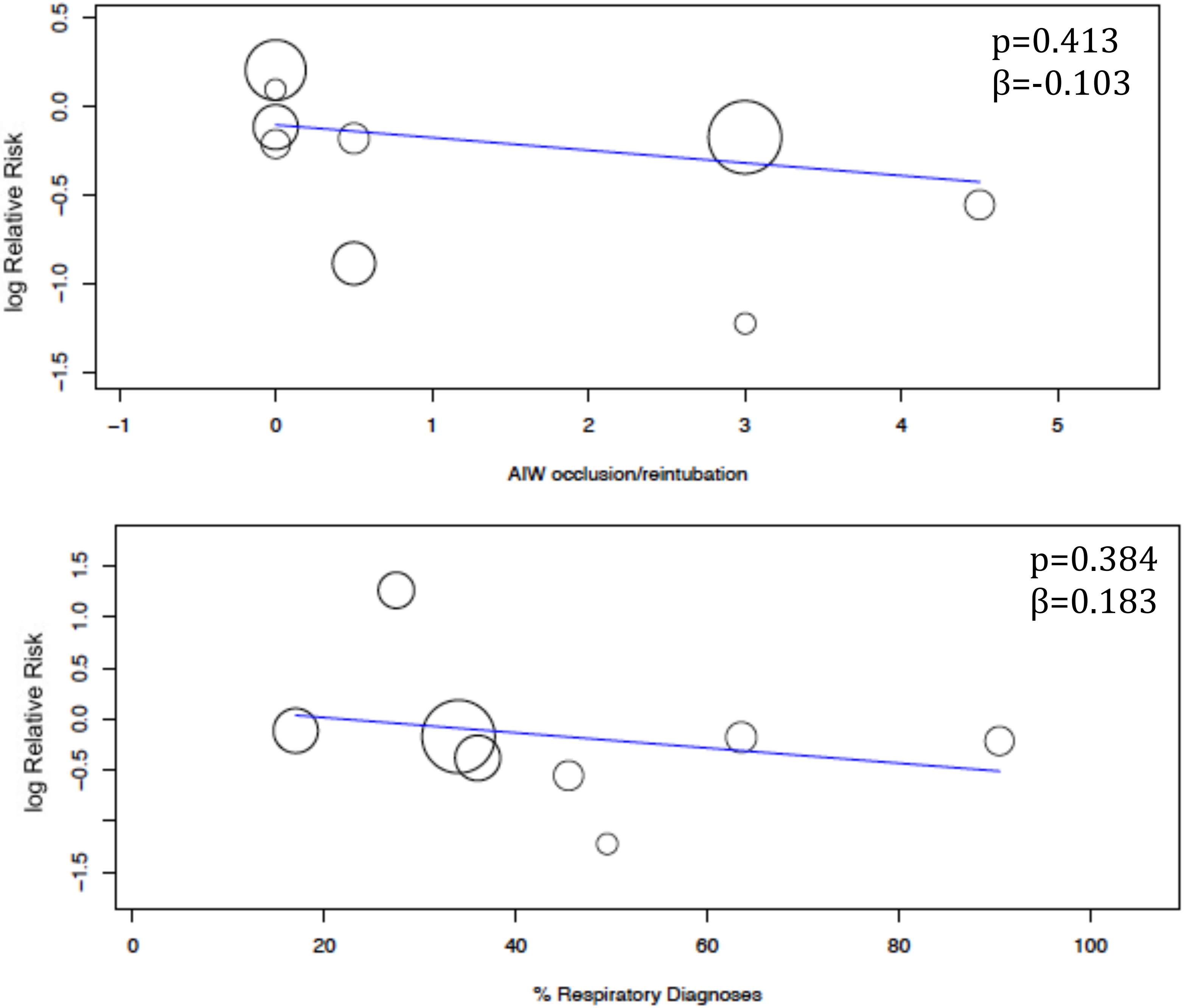
p=0.413
β=-0.103
log Relative Risk
0.5
0.0
−0.5
−1.0
−1.5
−1
0
1
2
3
4
5
AIW occlusion/reintubation
p=0.384
β=0.183
log Relative Risk
1.5
1.0
0.5
0.0
−0.5
−1.5
0
20
40
60
80
100
% Respiratory Diagnoses

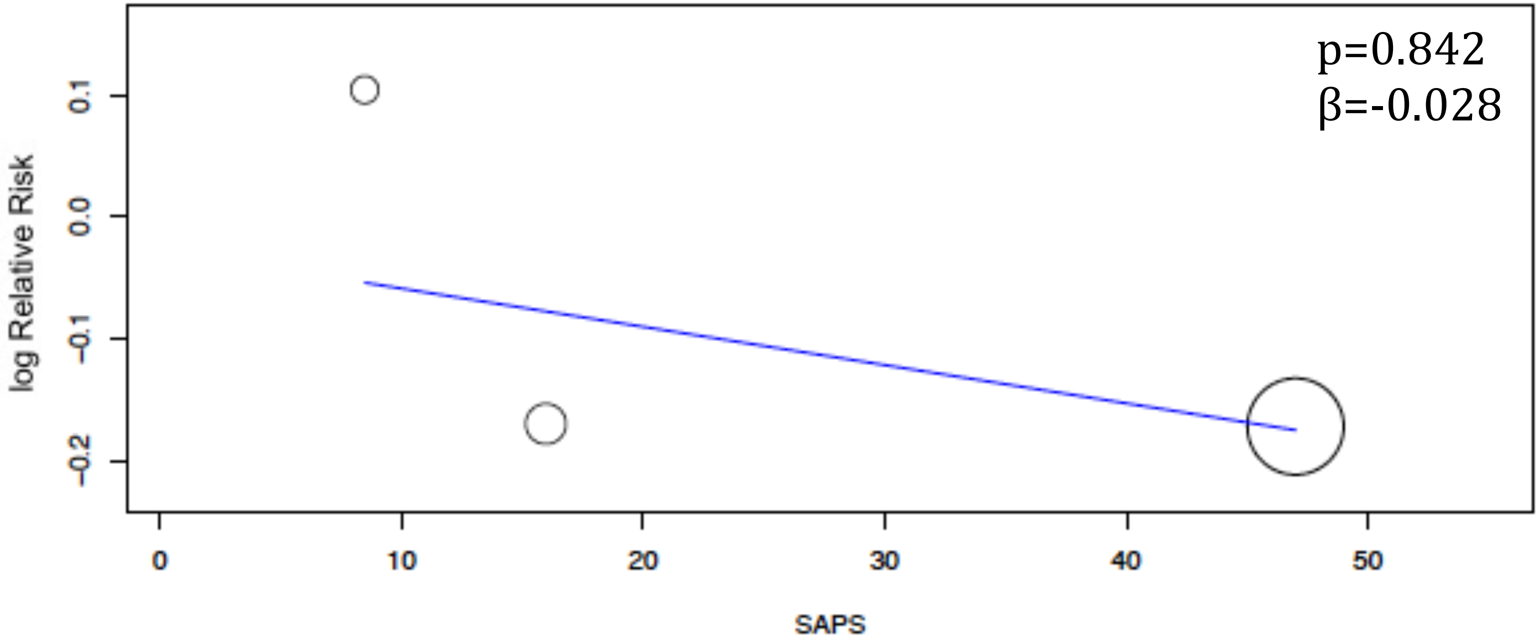
p=0.842
β=-0.028
log Relative Risk
0.1
0.0
−0.1
−0.2
0
10
20
30
40
50
SAPS

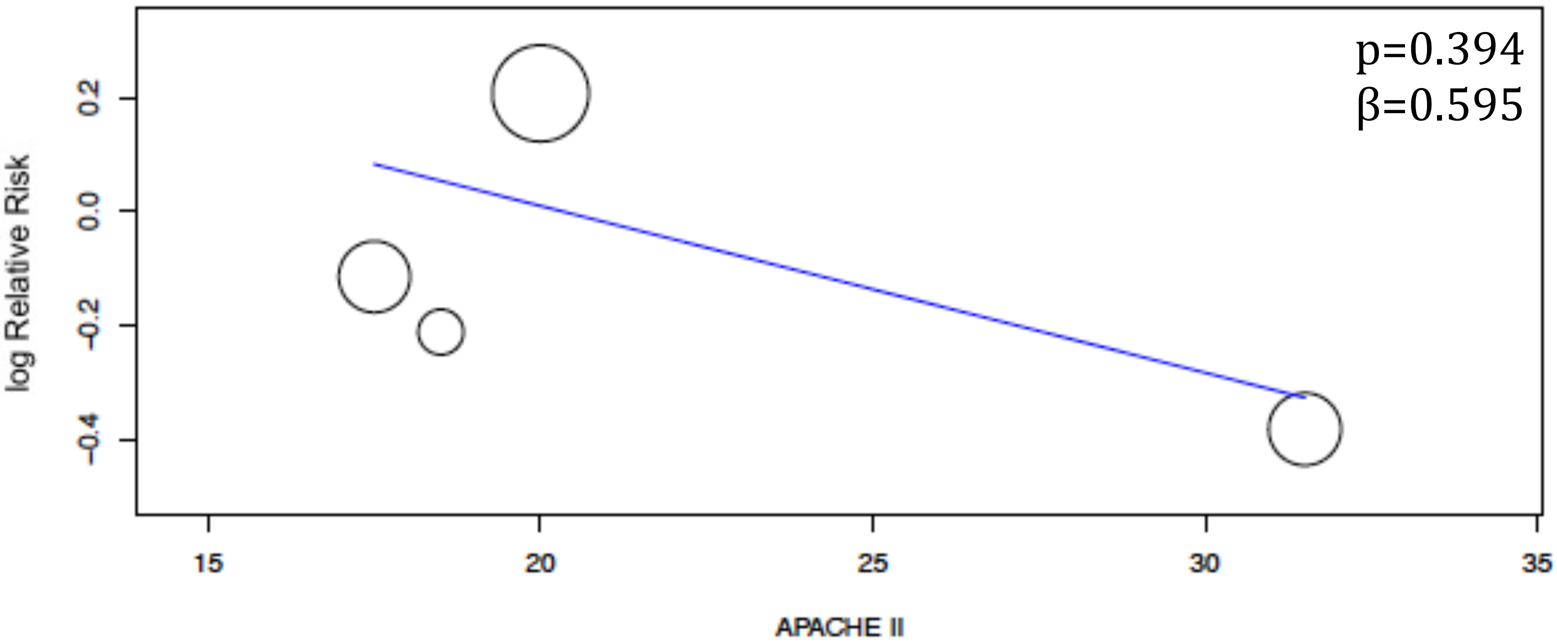
p=0.394
β=0.595
log Relative Risk
0.2
0.0
−0.2
−0.4
15
20
25
30
35
APACHE II

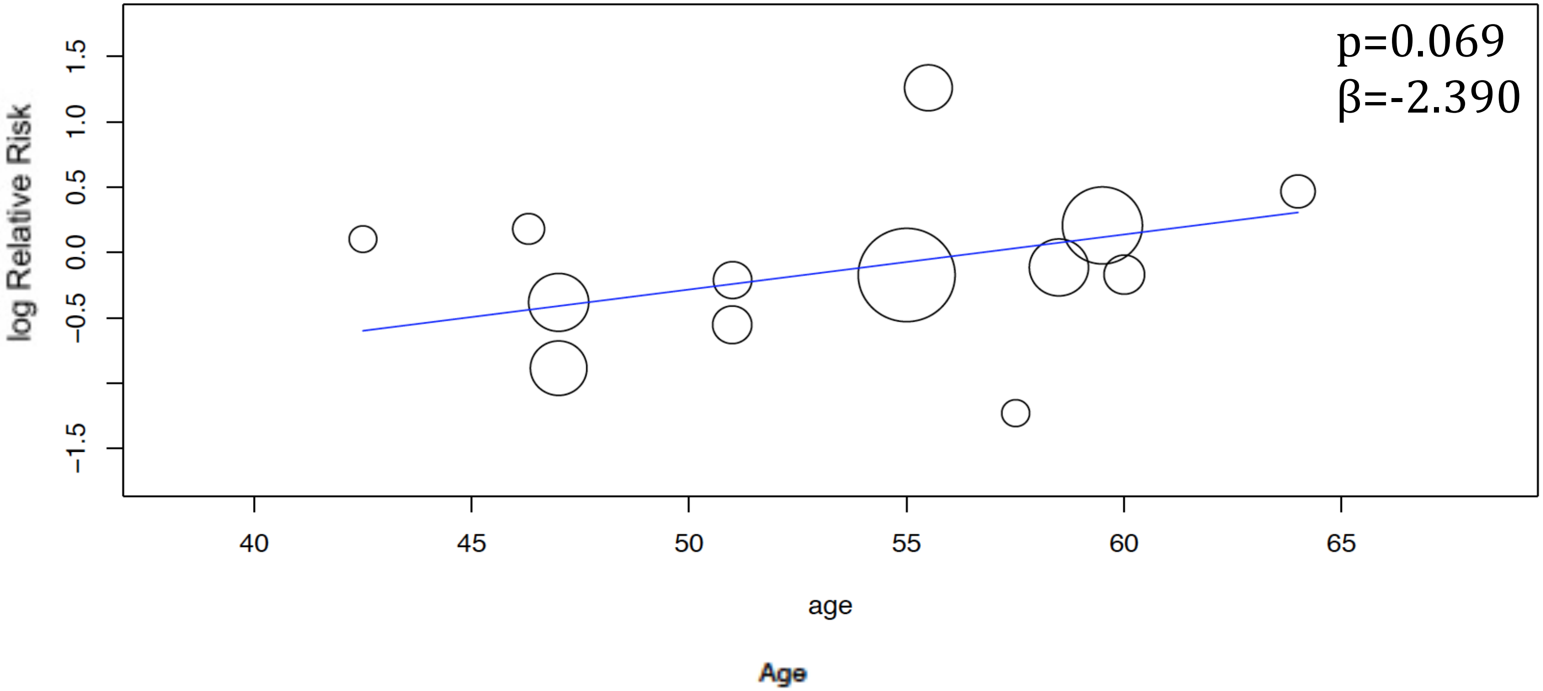
p=0.069
β=-2.390
log Relative Risk
−1.5
−0.5
0.0
0.5
1.0
1.5
40
45
50
55
60
65
age
Age

META-REGRESSION FOR MORTALITY

No significant relationships were found between mortality and duration of MV, VAP incidence, ICU LOS, AIW occlusion/reintubation, % respiratory diagnoses, SAPS, APACHE II and age.

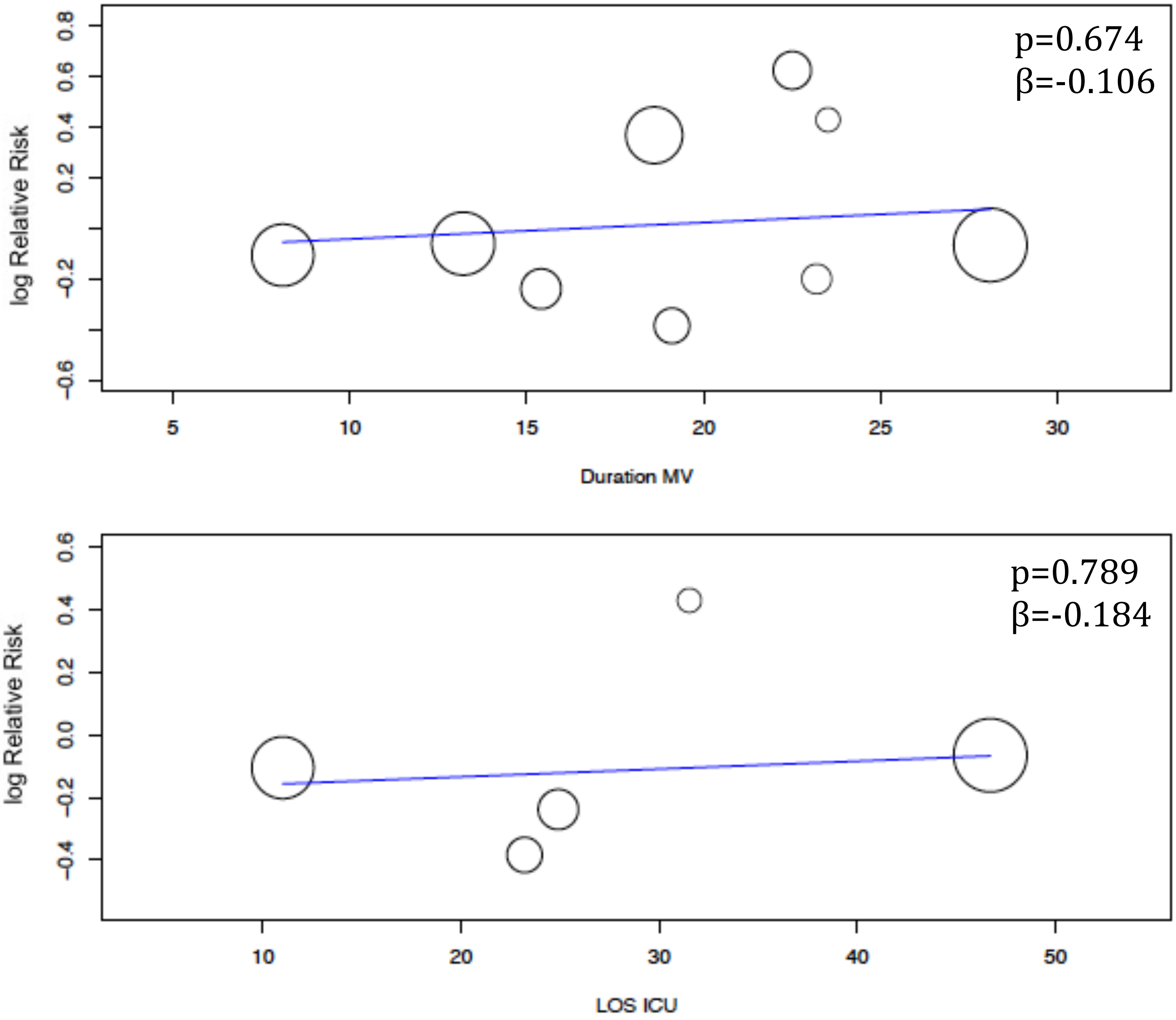

p=0.674
β=-0.106
log Relative Risk
0.8
0.6
0.4
0.2
-0.2
-0.6
5
10
15
20
25
30
Duration MV
p=0.789
β=-0.184
log Relative Risk
0.6
0.4
0.2
0.0
-0.2
-0.4
10
20
30
40
50
LOS ICU

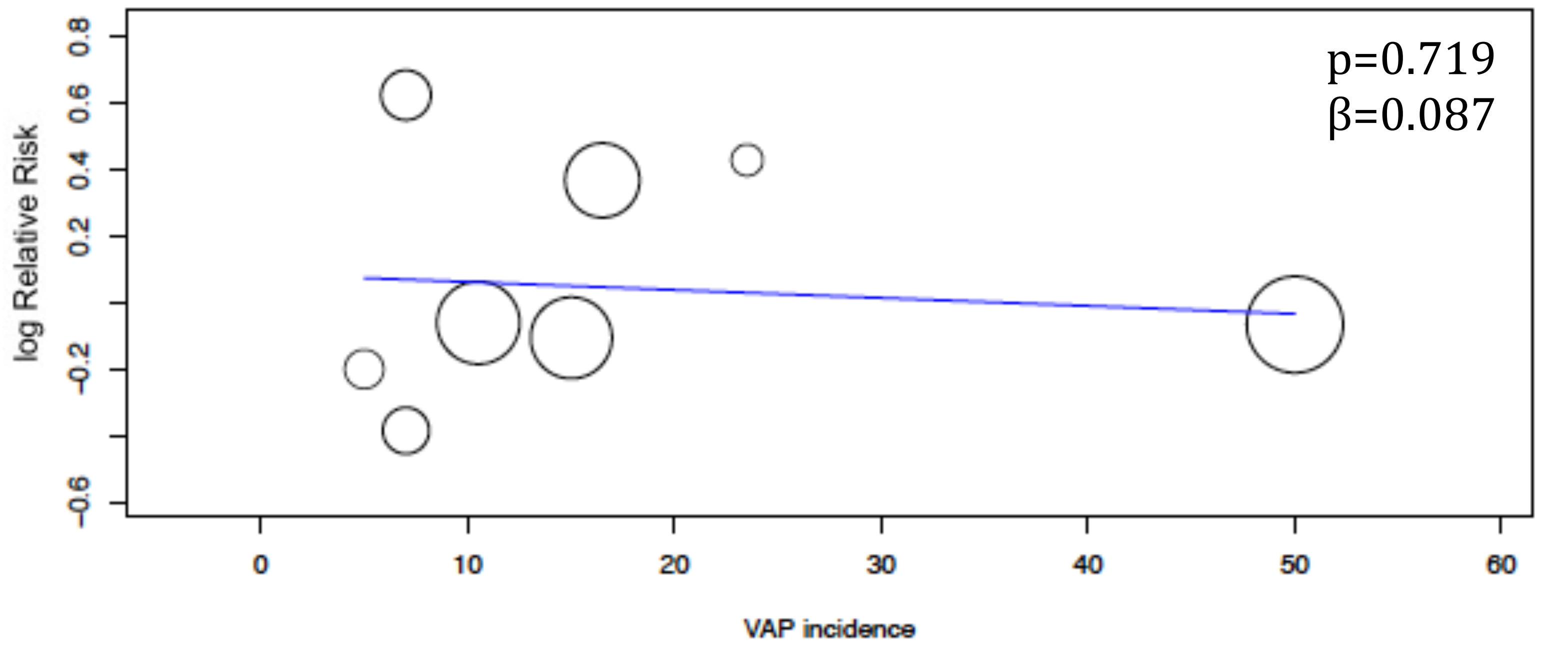
p=0.719
β=0.087
log Relative Risk
VAP incidence

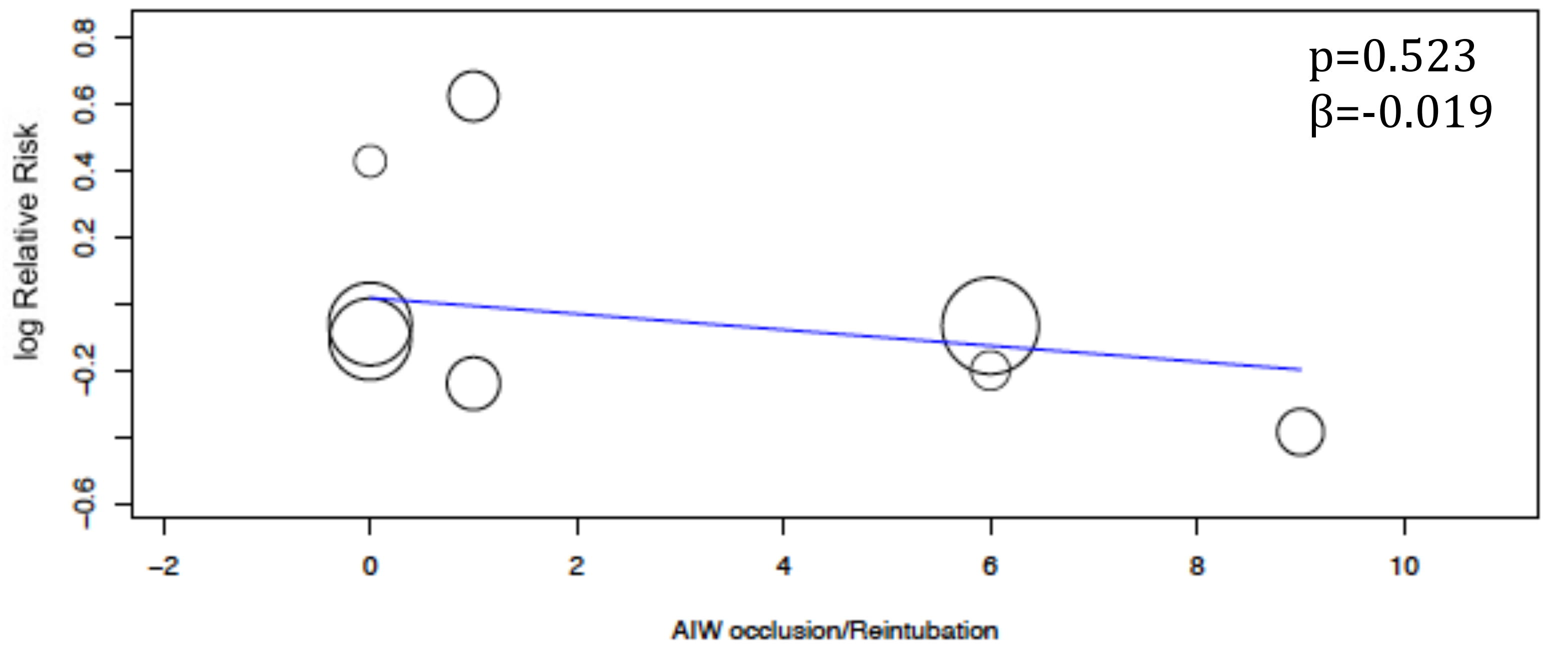
p=0.523
β=-0.019
log Relative Risk
AIW occlusion/Reintubation

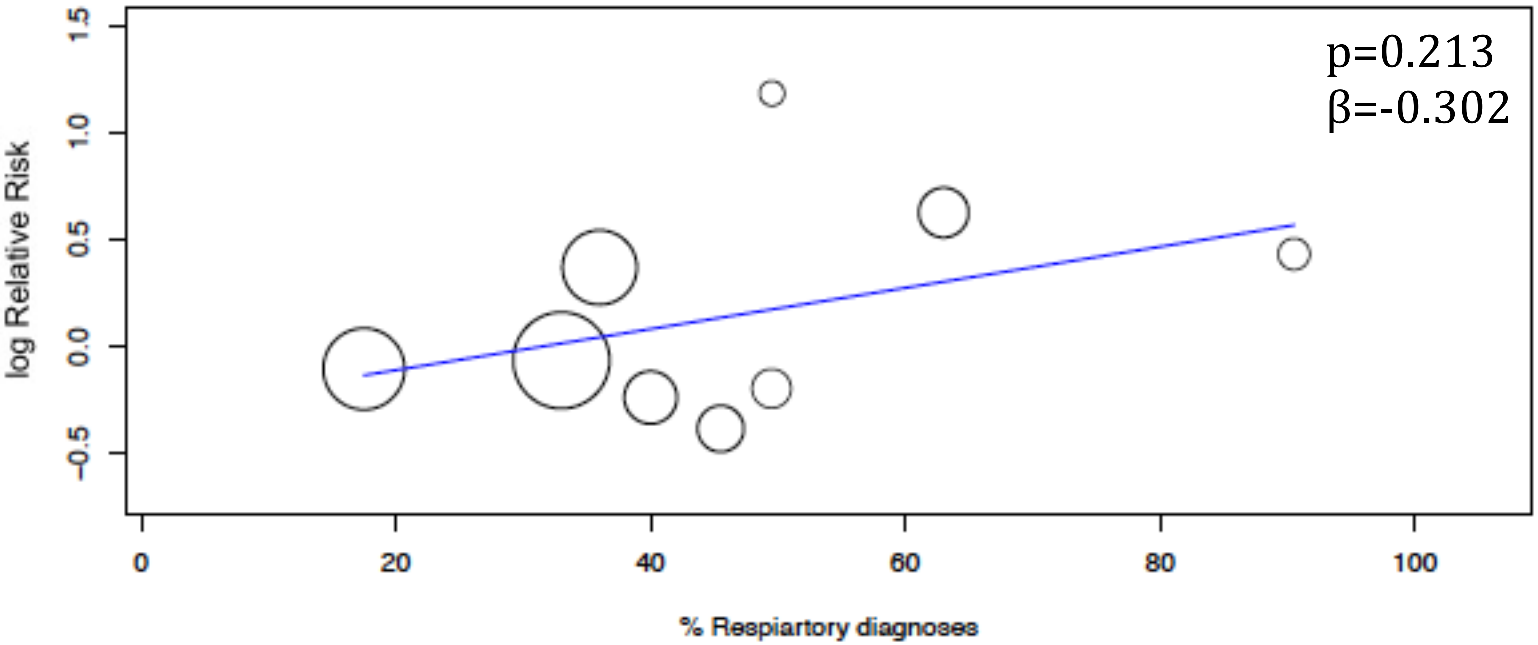
p=0.213
β=-0.302
log Relative Risk
−0.5
0.0
0.5
1.0
1.5
0
20
40
60
80
100
% Respiartory diagnoses

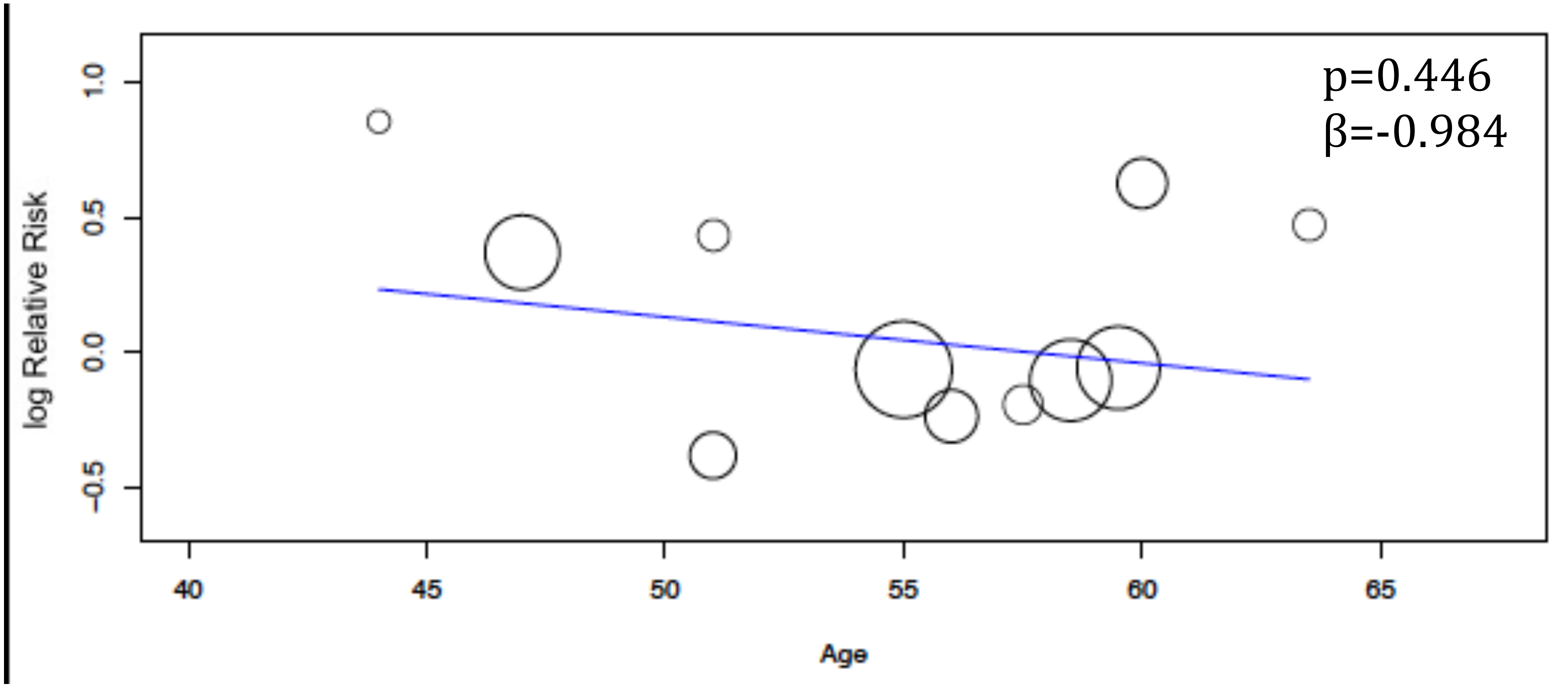
p=0.446
β=-0.984
log Relative Risk
−0.5
0.0
0.5
1.0
40
45
50
55
60
65
Age

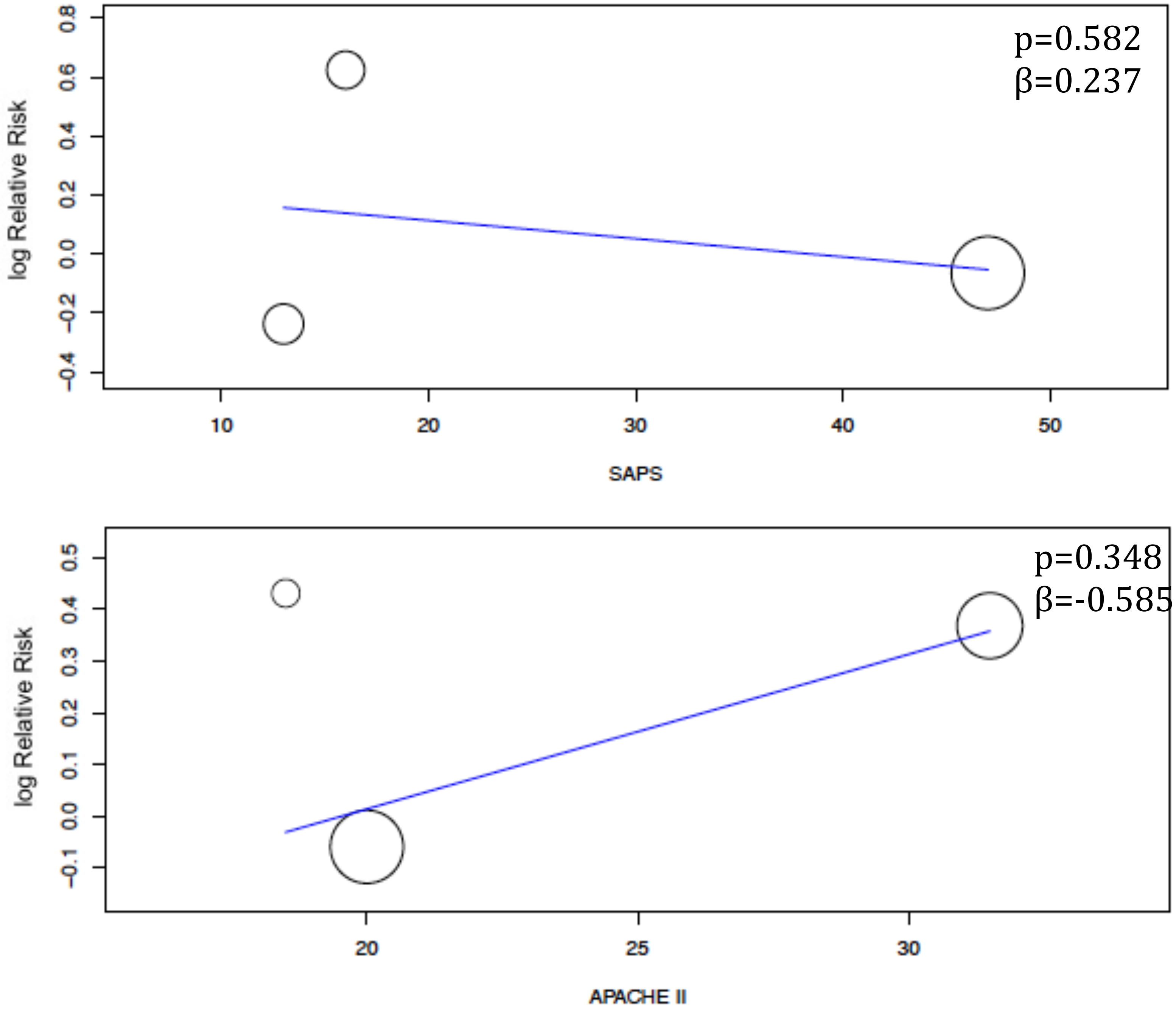
p=0.582
β=0.237
log Relative Risk
−0.4
−0.2
0.0
0.2
0.4
0.6
0.8
10
20
30
40
50
SAPS
p=0.348
β=-0.585
log Relative Risk
−0.1
0.0
0.1
0.2
0.3
0.4
0.5
20
25
30
APACHE II
