# Supplementary material for: Heat and moisture exchangers (HMEs) and heated humidifiers (HHs) in adult critically ill patients: a systematic review, meta-analysis and meta-regression of randomized controlled trials
Source: Crit Care. 2017 May 29;21:123. doi: 10.1186/s13054-017-1710-5 (PMC5447307; doi:10.1186/s13054-017-1710-5)
Supplement: Supplementary file 3 — Meta-analyses for hydrophobic and hygroscopic HME vs HH for Artificial airway occlusion, mortality and Pneumonia. (PDF 231 kb) [file 13054_2017_1710_MOESM3_ESM.pdf]

Artificial airway occlusion

Studies	Estimate (95% C.I.)	HME hydrophobic	HH
Martin 1990	17.469 (1.021, 298.935)	6/31	0/42
Misset 1991	1.733 (0.345, 8.707)	4/30	2/26
Roustan 1992	21.036 (1.253, 353.184)	9/55	0/61
Villafane 1996	3.187 (0.368, 27.577)	3/16	1/17
Kirton 1997	0.143 (0.007, 2.740)	0/140	3/140
**Overall (**	**2.990 (0.671, 13.327)**	**22/272**	**6/286**

Tau^2=1.385; Q (df=4)=7.810; I^2=48%; p=0.099
Test overall effect Z=3.234; p=0.151

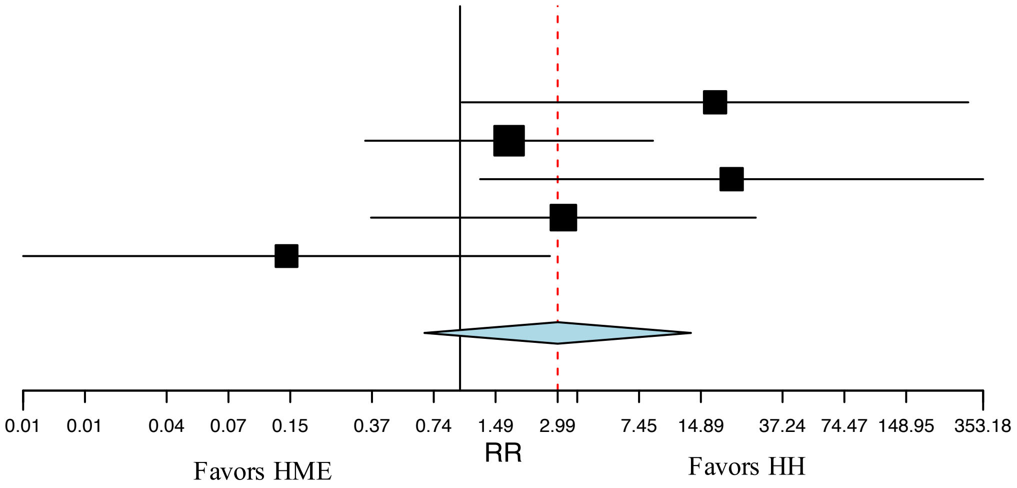

Weight:
Martin: 16.7%, Misset: 28.2%, Roustan:16.8%, Villafane:22.4%Kirton: 15.9%

Studies	Estimate (95% C.I.)	HME hydroscopic	HH
Kirkegaard 1987	5.000 (0.260, 96.127)	2/15	0/15
Daud 1991	0.645 (0.330, 1.258)	9/29	13/27
Dreyfuss 1995	3.435 (0.143, 82.813)	1/61	0/70
Branson 1996	1.100 (0.022, 54.407)	0/49	0/54
Boots 1997	0.977 (0.020, 48.099)	0/42	0/41
Hurni 1997	0.317 (0.013, 7.615)	0/59	1/56
Kollef 1998	0.902 (0.018, 45.194)	0/163	0/147
Lucchetti 1998	13.562 (0.745, 246.762)	3/15	0/30
Lacherade 2005	0.199 (0.023, 1.686)	1/185	5/184
boots 2006	1.005 (0.020, 50.402)	0/190	0/191
**Overall**	**0.767 (0.436, 1.348)**	**16/808**	**19/815**

Tau^2=0.000; Q (df=9)=78.325; I^2=0%; p=0.502
Test overall effect Z=0.489; p=0.624

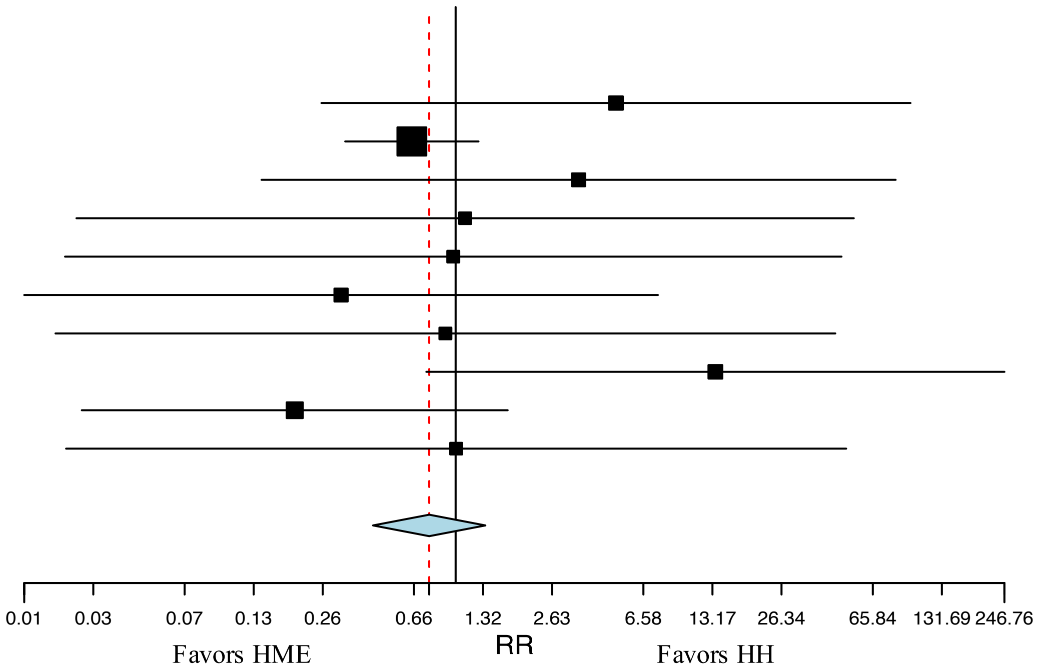

Weight:
Kirkegaard:3.6%, Daud:71%, Dreyfuss: 3.1%, Branson: 2%, Boots:2%, Hurni: 3.1%, Lucchetti: 3.8%, Lacherade: 7%,boots: 2.1%

Pneumonia

Studies	Estimate (95% C.I.)	HME hydrophobic	HH
Martin 1990	0.293 (0.058, 1.491)	2/31	8/42
Roustan 1992	0.578 (0.181, 1.843)	5/55	9/61
Kirton 1997	0.413 (0.188, 0.907)	10/140	22/140
**Overall**	**0.431 (0.235, 0.790)**	**17/226**	**39/243**

Tau^2=0.000; Q (df=2)=0.473 ; I^2=0%; p=0.789
Test overall effect Z=2.529; p=0.011

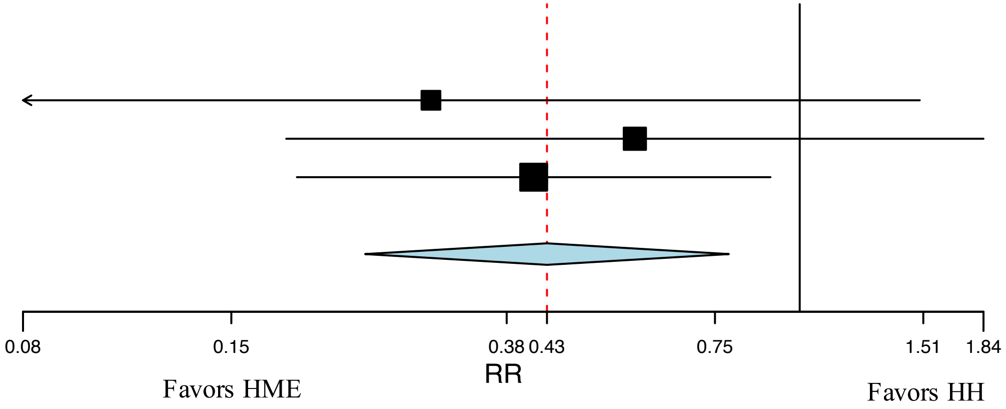

Weight:
Martin:13.8%,
Roustan:27.2%,
Kirton:59%

Studies	Estimate (95% C.I.)	HME hydroscopic	HH
Dreyfuss 1995	0.845 (0.276, 2.589)	6/61	8/70
Branson 1996	1.109 (0.213, 5.768)	3/49	3/54
boots 1997	0.810 (0.247, 2.653)	6/42	7/41
Kollef 1998	0.892 (0.420, 1.894)	15/163	15/147
Memish 2001	0.683 (0.325, 1.433)	14/123	19/120
Diaz 2002	1.600 (0.424, 6.031)	8/23	5/20
Lachede 2005	0.842 (0.532, 1.333)	47/185	53/184
Lorente 2006	3.527 (1.386, 8.977)	21/53	8/51
Boots 2006	1.230 (0.705, 2.147)	32/190	27/191
**Overall**	**1.049 (0.776, 1.418)**	**152/889**	**145/878**

Tau^2=0.039; Q (df=8)=9.832 ; I^2=18%; p=0.277
Test overall effect Z=0..276; p=0.076

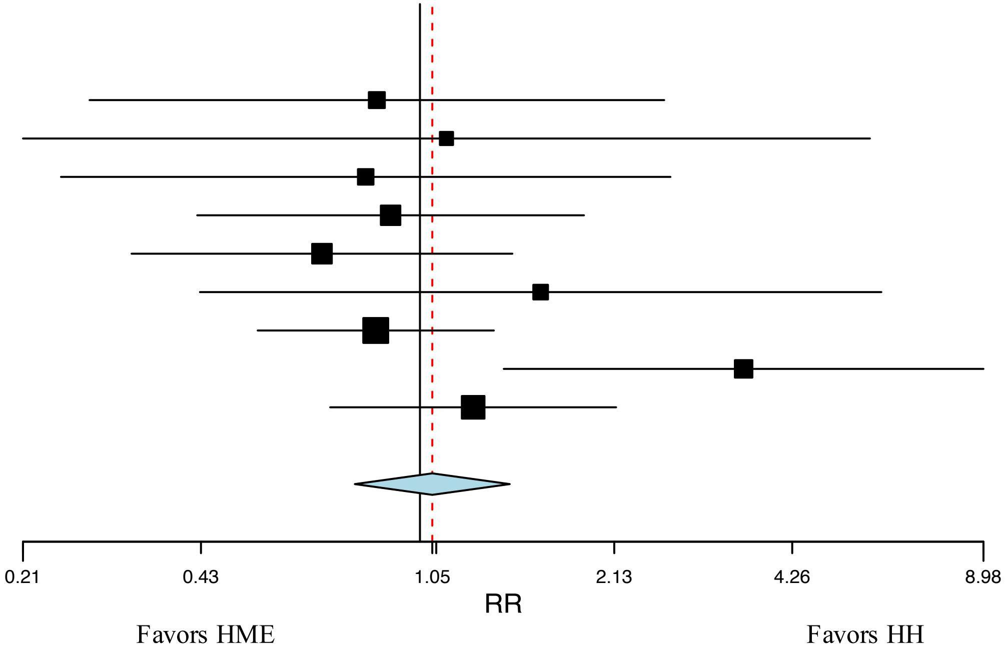

Weight:
Dreyfuss: 6.5%,
Branson: 3.1%, boots: 5.8%, Boots: 19.2%,
Diaz: 4.7%, 1.3%,
Kollef: 12.7%,
Lacherade: 25.3%,
Memish: 13.1%,
Lorente: 8.9%

Mortality

Studies	Estimate (95% C.I.)	HME hydrophobic	HH
Martin 1990	0.822 (0.277, 2.438)	7/31	11/42
Roustan 1992	0.681 (0.277, 1.675)	10/55	15/61
**Overall**	**0.735 (0.368, 1.471)**	**17/86**	**26/103**

Tau^2=0.000; Q (df=1)=0.068 ; I^2=0%; p=0.795
Test overall effect Z=0.711; p=0.477

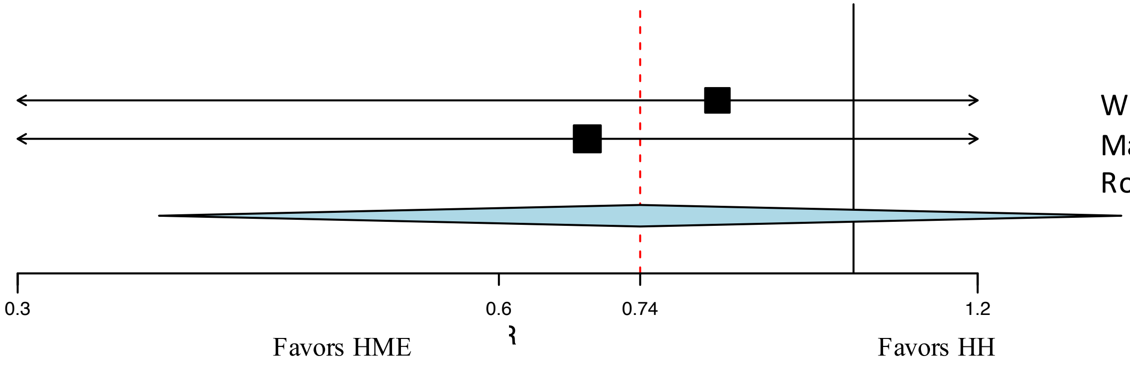

Weight:
Martin 40.6%,
Roustan: 59.4%

Studies	Estimate (95% C.I.)	HME hydroscopic	HH
boots 1997	1.542 (0.401, 5.922)	6/42	4/41
Boots 2006	0.943 (0.570, 1.558)	37/190	39/191
Daud 1991	3.261 (0.597, 17.806)	6/29	2/27
Diaz 2002	1.600 (0.424, 6.031)	8/23	5/20
Dreyfuss 1995	1.867 (0.809, 4.310)	17/61	12/70
Hurni 1997	0.788 (0.358, 1.736)	17/59	19/56
Kirkegaard 1987	2.364 (0.361, 15.455)	4/15	2/15
Kollef 1998	0.901 (0.540, 1.502)	40/163	39/147
Lacherade 2005	0.937 (0.609, 1.443)	61/186	63/184
Memish 2001	1.446 (0.826, 2.530)	40/123	30/120
**Overall**	**1.092 (0.879, 1.358)**	**236/891**	**215/871**

Tau^2=0.000; Q (df=9)=7.374 ; I^2=0%; p=0.598
Test overall effect Z=0.505; p=0.445

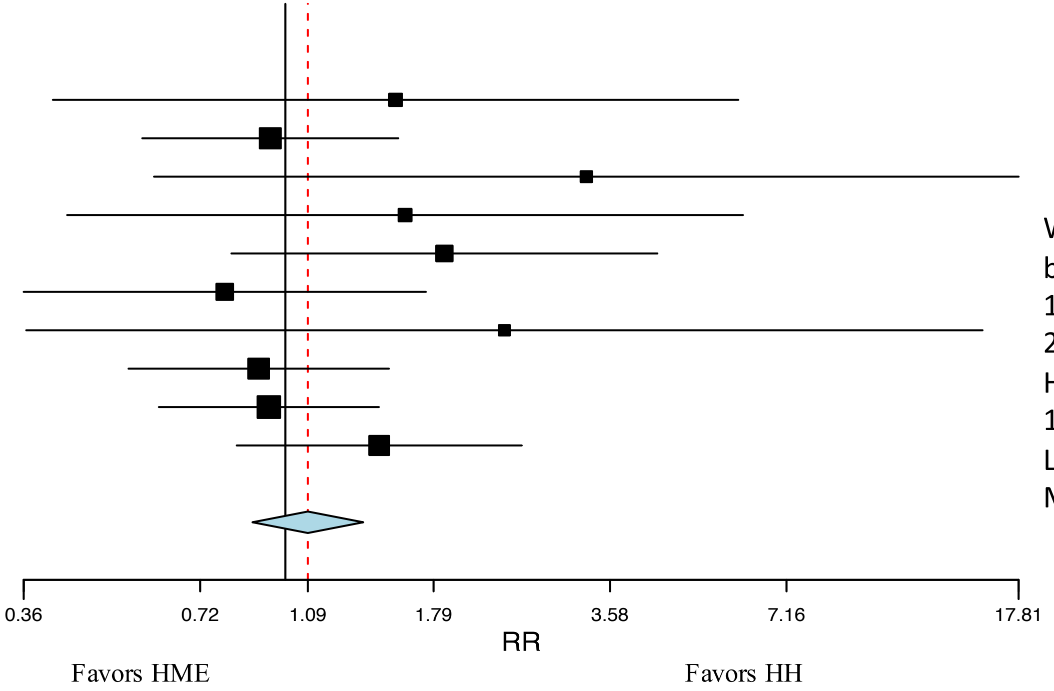

Weight
boots: 2.6%, Boots: 18.7%, Daud: 1.6%, Diaz: 2.7%, Dreyfuss: 6.7%, Hurni: 7.6%, Kierkegaard: 1.3%, Kollef: 18.1%, Lacherade: 25.4%, Memish: 15.1%
